# Learning efficient representations of environmental priors in working memory

**DOI:** 10.1371/journal.pcbi.1011622

**Published:** 2023-11-09

**Authors:** Tahra L. Eissa, Zachary P. Kilpatrick

**Affiliations:** 1 Department of Applied Mathematics, University of Colorado Boulder, Boulder, Colorado, United States of America; 2 Institute of Cognitive Science, University of Colorado Boulder, Boulder, Colorado, United States of America; Brown University, UNITED STATES

## Abstract

Experience shapes our expectations and helps us learn the structure of the environment. Inference models render such learning as a gradual refinement of the observer’s estimate of the environmental prior. For instance, when retaining an estimate of an object’s features in working memory, learned priors may bias the estimate in the direction of common feature values. Humans display such biases when retaining color estimates on short time intervals. We propose that these systematic biases emerge from modulation of synaptic connectivity in a neural circuit based on the experienced stimulus history, shaping the persistent and collective neural activity that encodes the stimulus estimate. Resulting neural activity attractors are aligned to common stimulus values. Using recently published human response data from a delayed-estimation task in which stimuli (colors) were drawn from a heterogeneous distribution that did not necessarily correspond with reported population biases, we confirm that most subjects’ response distributions are better described by experience-dependent learning models than by models with fixed biases. This work suggests systematic limitations in working memory reflect efficient representations of inferred environmental structure, providing new insights into how humans integrate environmental knowledge into their cognitive strategies.

## Author summary

Working memory is known to play an important role in cognition, allowing us to maintain information in our memory for short periods without a constant stimuli. However, humans display limitations in working memory, such as recalling certain stimuli more frequently and accurately than others. We propose that these recall biases are based on our experience of common stimuli in our environment and driven by goal of efficiently reducing error by remembering common stimuli with more accuracy than rare stimuli. Here, we develop a model that updates an observer’s beliefs about the statistics of stimuli in an environment based on experience, biasing working memory recall such that common stimuli are remembered better. We then show that most human subjects’ responses from a previously published working memory task are better matched to a model that learns in an experience-dependent way compared to models with fixed biases. Finally, we identify a plausible neural mechanism for environmental experience-updating to show how the brain could implement this efficient strategy.

## Introduction

Traditional descriptions of working memory, a core feature of cognition [1], conceive of a system that takes in, maintains, and computes information over short timescales without a constant source of input. Knowing the limitations of this system can help identify its role in cognition [2] and provide a bridge to developing relevant neural theories. The limits and biases of working memory can be measured by the statistics of recall errors after a delay, for instance, in a visual delayed response task [3]. In these tasks, humans are asked to recall object features, such as location, color, or shape, a short time after presentation [2, 4–6]. When feature values lie on a continuum, subject responses do as well, giving finely resolved measurements of the direction and magnitude of errors on each trial [7, 8]. For example, people’s responses on delayed-response tasks often exhibit error magnitudes that increase roughly linearly with time, comparable to the variance of a diffusion process [9, 10], providing a metric that can guide neural theories for working memory.

Complementary to behavioral studies of working memory, theories describing how the brain encodes information over short periods of time provide mechanistic insight. One well-validated theory associates remembered stimulus values with persistent neural activity in recurrently coupled excitatory neurons that are preferentially tuned to the target values [11]. Broadly tuned inhibitory neurons driven by excitation stabilize this activity into a localized structure called an activity *bump* [12, 13]. Variability in neural tuning and synaptic connectivity can cause this activity bump to wander about feature space, causing trial-by-trial errors and biases often perceived as limitations to the system [14–16]. For example, delayed estimates may exhibit serial bias, whereby stimulus values from previous trials may attract or repel the retained memory of the most recent stimulus value [17]. Analogous attractive biases emerge when subjects retain the values of multiple stimuli within a single trial [18]. Additionally, subjects may exhibit systematic biases that include preferences for focal colors [16, 19], orientations [20] and cardinal directions [21].

While biases are often considered reflections of suboptimality, they can be advantageous when reflecting the structure of the environment or sequences of stimuli the subject might see [22, 23]. There is ample evidence that working memory can be trained, and such biases may be the result of long-term learning [24]. Mechanistically, systematic biases in stimulus coding or delayed estimates could emerge from heterogeneities in synaptic connectivity, so collective neural activity is biased to specific network conformations [25]. Such heterogeneity could also be reflected in variations in the sensitivity of individual neurons’ stimulus feature tuning [6, 26, 27]. Heterogeneity in the spatial organization of synaptic connectivity can reduce error by maintaining representations that are less susceptible to noise perturbations [28–30]. Thus, if synaptic heterogeneity reflects the learned or expected distribution of stimulus values, recall of common features could be less error prone, improving cognitive efficiency [31].

Since certain stimulus features may be overrepresented in the natural world (e.g., green/brown colors are more common in a forest; see also [32, 33]), we propose that subjects’ systematic biases could result from learning the natural distribution of specific features of the environment, which modulates synaptic connectivity to produce representation biases. Here, we model the effects of environmental feature distributions on delayed estimation in neural circuit models and their low-dimensional reductions, considering both models with network connectivity that is fixed and those shaped by long-term plasticity. We compare these results to human behavior and find that most subjects exhibit strategies best described by learning models, supporting the hypothesis that long-term representation biases reflect the learning of environmental structure.

## Results

**Fig 1:**
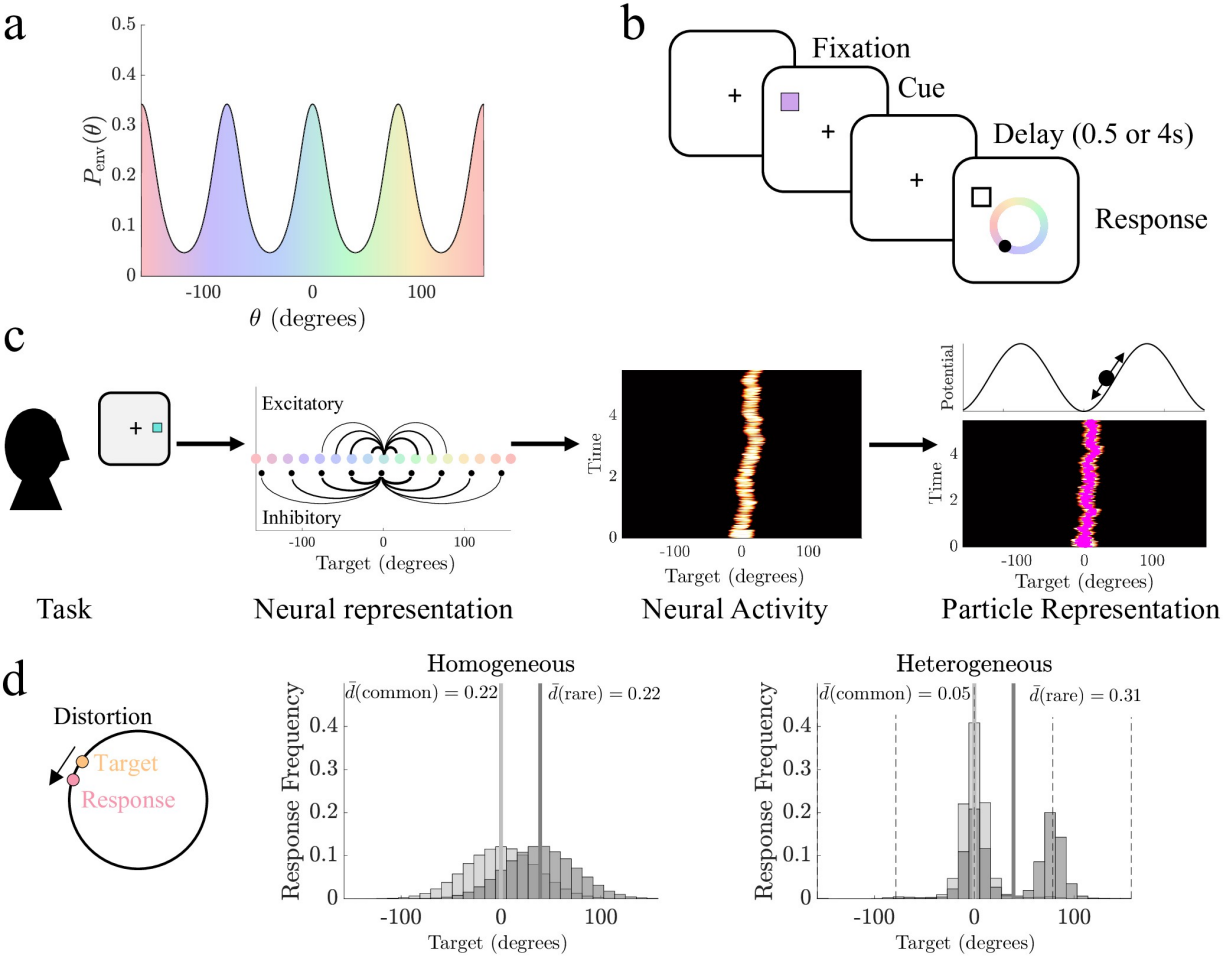
Heterogeneity in the distribution of environmental features is reflected in delayed estimates. **a**. Natural environments that include overrepresented features, such as certain colors, are described by heterogeneous priors of feature distributions with peaks at the overrepresented features. **b**. Schematic of the delayed estimation task, which requires subjects to remember a target feature (e.g., color) and report it following a short delay period. **c**. The remembered target feature is represented neuromechanistically by a subpopulation of stimulus-specific excitatory neurons with local recurrent excitatory and broad inhibition. The target representation is retained as a bump of sustained neural activity wandering stochastically during the delay. Bump dynamics can be projected to a particle model describing its stochastically evolving position: Spatial heterogeneity in synaptic connectivity is inherited by the particle model as a nontrivial energy landscape with attractors corresponding to regions of enhanced excitation. **d**. Distortion, the circular distance between the target and responses, is influenced by synaptic heterogeneity. In homogeneous networks, response errors at common environmental targets (*θ* = 0, light grey) and rare targets (*θ* = 45, dark grey) are equivalent, giving the same local mean distortion ($\bar{d}\left(\theta \right)$). With synaptic heterogeneity matched to the environmental prior *P*_env_(*θ*), errors are reduced near common stimulus feature values (dashed lines). Parameters used as listed in Methods Table 1.

We begin with the premise that features of natural environments are distributed such that particular values that are overrepresented and thus, statistically more likely to occur as samples (Fig 1A). Such parametric distributions could take on general forms [27], but for illustration, we assume a parametric prior that is periodic with peaks (dips) at common (rare) stimulus values

$$\begin{array}{c} P_{\text{env}}\left(\theta \right)=e^{A\;\text{cos}(m\theta )}, \end{array}$$

where *θ* corresponds with a particular feature value described on a ring (e.g., one feature dimension wraps periodically), *A* describes the amplitude, and *m* describes the number of peaks in the probability distribution. This periodic function resembles the color biases displayed by humans in [16] as well as cardinal bias common to angle and direction estimates [20, 21]. Note, unless otherwise stated, all subsequent results assume that *m* = 4, so the peaks are centered at cardinal angles of *θ*, given in radians in formulas, but plotted in degrees in figures for readability.

Our models describe the maintenance of estimates of continuous features [34, 35], arising in tasks where an observer is briefly shown a number of items and, after a delay, probed about remembered stimulus feature values (e.g., location, orientation, or color). These models allow us to speculate on how the environmental priors impact (and potentially bias) how stimulus feature values are remembered (Fig 1B). To illustrate, we focus on examples in which subjects recall colors, though equivalent results can be produced for models of orientation and location recall. Our models are motivated by previous observations that show human performance on delayed estimation tasks degrades over time, such that response variance increases roughly linearly, suggesting a diffusive process drives memory errors [9]. Such diffusive degradation of a stimulus estimate has been modeled in neural circuits as a localized region of persistent activity (bump) that stochastically wanders feature space due to neural and synaptic fluctuations [11, 36]. Activity bumps emerge from strong stimulus-tuned recurrent excitation paired with broad stabilizing inhibition, which generates self-sustained activity [12]. Spatial variation in synaptic connectivity can shape the preferred locations (attractors) of the bump, introducing drift toward attractors [28, 29, 37, 38].

Since the location of the activity bump is a proxy for the remembered stimulus feature value [14, 15], we can simplify our analysis of the impact of activity bump fluctuations by considering low-dimensional models that describe the bump as a particle stochastically moving through an energy landscape (Fig 1C). The (negative) gradient of this energy landscape determines the direction of the drift in the stored estimate [28]. Moreover, asymptotic methods can be used to link the synaptic heterogeneity of neural circuits [39] to a tractable model of the bump position’s low-dimensional dynamics [38, 40]. Energy landscapes can be updated to represent an observer’s current estimate of the environmental feature distribution *P*_env_(*θ*) (see Methods) and can be more easily fit to response data than neural circuit models [14, 16, 23, 41], providing a tractable model for studying the origins of systematic biases in working memory.

We compute our models’ average error between a true target value *θ* and its estimate as the mean distortion $\bar{d}\left(\theta \right)$, the circular distance between the target and responses. Overall error across all target values is computed as the total mean distortion ${\bar{d}}_{\text{tot}}=\int_{-\pi }^{\pi }\bar{d}\left(\theta \right)P_{\text{env}}\left(\theta \right)d\theta$ [15, 29]. Thus, when synaptic connectivity (and the corresponding energy landscape) is aligned with the environmental prior *P*_env_(*θ*), the mean distortion is reduced at common target feature values $\bar{d}\left(\theta_{\text{common}}\right)$ but increased for rare values $\bar{d}\left(\theta_{\text{rare}}\right)$. In contrast, purely distance-dependent synaptic connectivity (and a flat energy landscape) produces response distributions and mean distortion that are similar for common and rare target feature values (Fig 1D), making mean distortion a useful metric for quantifying error with respect to changes in synaptic connectivity.

Combining analysis of the energy landscapes with our distortion metric, we now systematically consider the impacts of environmental stimulus distributions on working memory responses, which can guide our understanding of how expectations about the environmental prior can be learned from experience and how these expectations can lead to more efficiently retained memories.

### Energy landscapes shape recall distortion

#### Uniform stimulus priors

**Fig 2:**
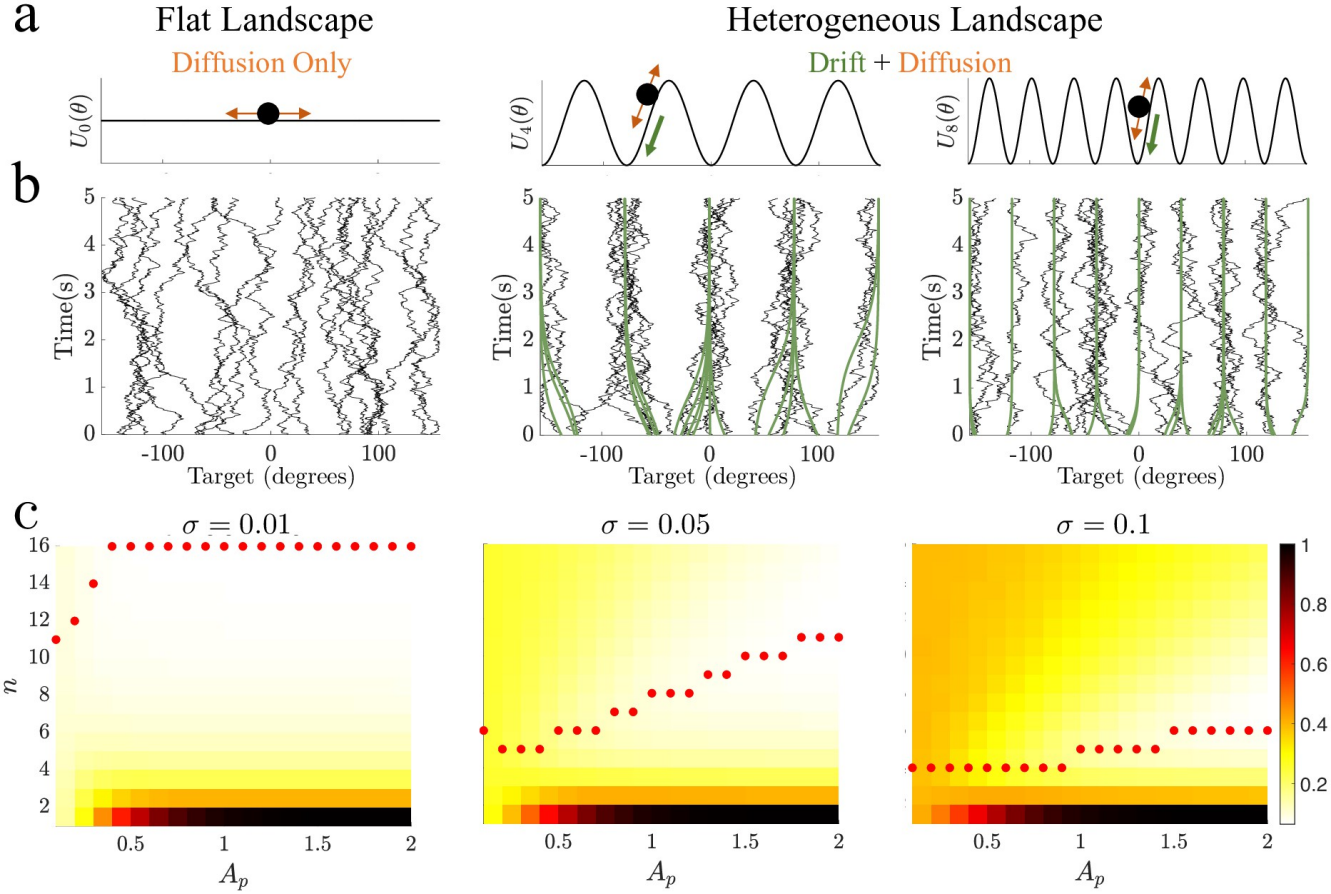
Particles in heterogeneous landscapes are drawn toward attractors. **a**. Schematics of the flat (homogeneous) landscape, with only diffusion, and heterogeneous landscapes, with potential-driven drift and diffusion. **b**. Example of particle trajectories in the flat and heterogeneous energy landscapes in **a**, sampled from a uniform environmental distribution. In the flat energy landscape, particle motion is driven purely by diffusion. In the heterogeneous energy landscapes, the particle drifts toward attractors over time but diffusion can cause the particle to “jump” wells. Drift-only process shown in green for comparison. Parameters: *A*_*p*_ = 0, 1, *n* = 4, 8 **c**. Total mean distortion as amplitude and number of wells was varied for delay of *T*_Delay_ = 5s and three diffusion values (*σ*). Optimal particle model identified based on minimum mean distortion (red dots). Parameters not listed for all sub-figures are in in Methods Table 1.

We consider a particle model that describes the stochastically evolving estimate of the target feature value in an energy landscape that can incorporate bias, introduced by breaking the symmetry of continuous attractor models of delayed estimation [42]. This low-dimensional model can be derived asymptotically from the stochastic evolution of the position *θ*(*t*) of an activity bump that encodes the estimate and the information about the prior in its network connectivity (see Methods). An energy landscape that reflects an observer’s long-term estimate of the periodically-varying environmental prior *P*_env_(*θ*) can be generated as

$$\begin{array}{c} U\left(\theta \right)=-A_{p}\;\text{cos}\left(n\theta \right), \end{array}$$ (1)

where *A*_*p*_ describes the well amplitude and *n* is the number of attractors (each located at the believed common environmental feature values). This simple form for *U*(*θ*) allows us to probe how the alignment of the energy landscape to the true environmental distribution shapes an observer’s distortion and produces response biases (Fig 2A).

**Table 1:** Parameter values for particle models.

Variable	Value
*σ*	0.05
*A*_*p*_	1
*n*	4
*T*_Delay_	5
*β*	8
*h*	0.25
*s*	5

The movement of the particle through this landscape evolves according to the stochastic differential equation

$$\begin{array}{c} d\theta \left(t\right)=-U^{\prime }\left(\theta \left(t\right)\right)dt+\sigma dW\left(t\right), \end{array}$$ (2)

where the particle evolves in response to the systematic drift induced by the energy landscape *U*(*θ*) and dynamic fluctuations generated by the Wiener process *W*(*t*). Considering a particle model with a flat energy landscapes (*A*_*p*_ ≡ 0), memory of the target stimulus feature evolves according to pure diffusion during the delay period. In contrast, particles evolving along non-trivial energy landscapes (*A*_*p*_ > 0) are biased toward the periodically placed attractors at *θ* = ±(*j*/*n*)*π* (*j* = 0, …, *n* − 1) (Fig 2B). See also [29, 43] for a detailed account of the *effective diffusion* which can be approximated in a diffusing particle model with a periodic energy landscape.

We first quantify the total mean distortion ${\bar{d}}_{\text{tot}}$ of responses from particle models encoding stimuli from a uniform environmental prior. Even given a uniform prior, delayed estimates can be improved due to the stabilizing effects of local attractors that mitigate the wandering from diffusion [28, 29, 44]. However, distortion of the target estimate is also enhanced by the introduction of drift induced by energy landscapes with attractors of varying the strength and number of attractors (Fig 2C). As diffusion increased, total distortion was reduced more by considering energy landscapes with fewer attractors, increasing the strength of energy barrier between attractors and the perturbation needed for particles to ‘jump’ between them (S1 Fig). Since longer delay times increase the possibility of these jumps, total distortion is best reduced in these cases by having fewer attractors, increasing the energy barrier between them. In this case, the reduction of effective diffusion due to strongly quantizing the space of possible particle positions counteracts the local increases in distortion that can arise due to the strong drift of particles starting close to the saddle [29, 43].

#### Heterogeneous stimulus priors

**Fig 3:**
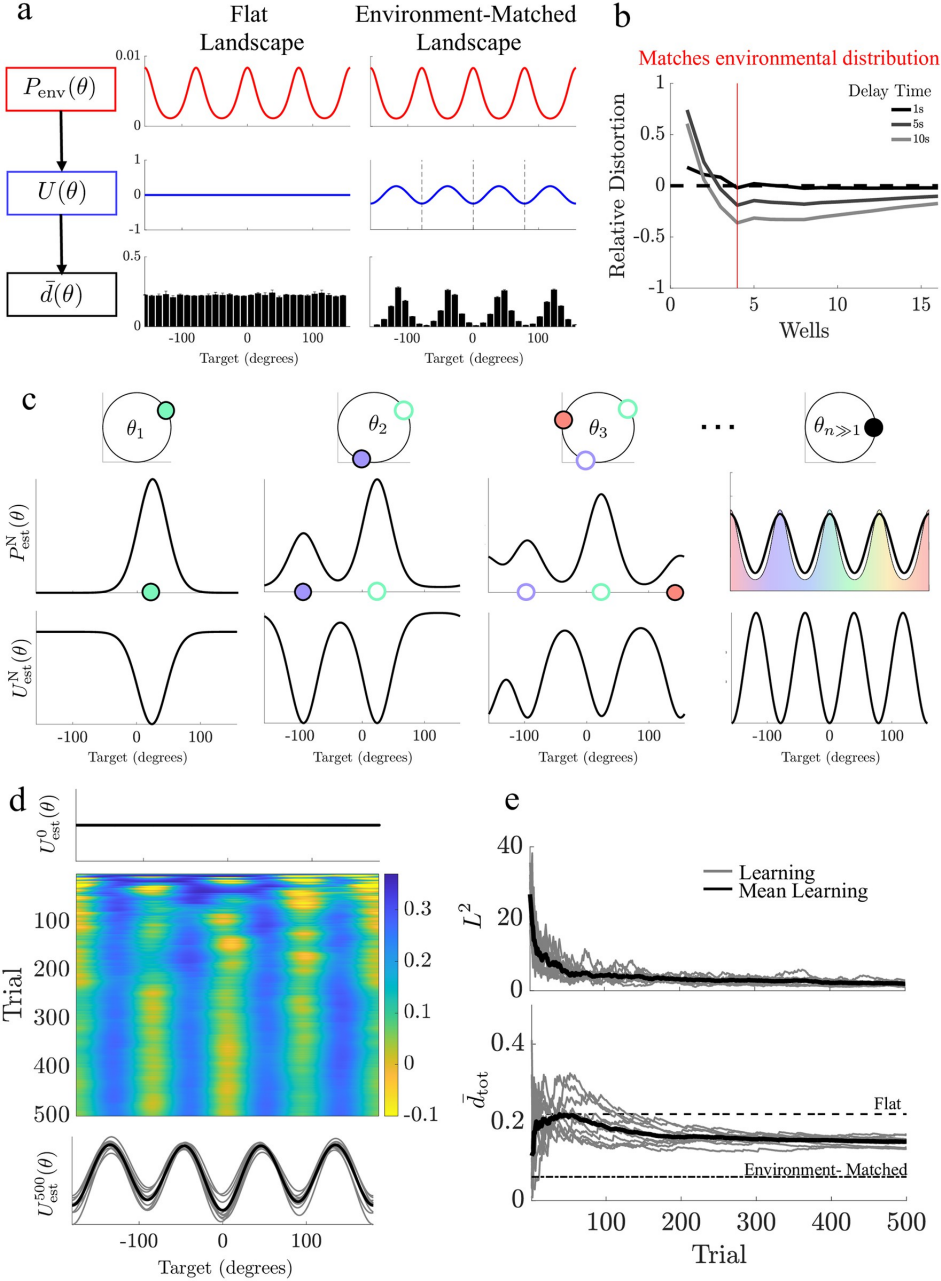
Distortion is reduced when energy landscapes match the environmental prior. **a**. Top: a heterogeneous environmental distribution (*P*_env_(*θ*)) passes through a heterogeneous energy landscape (*U*(*θ*)) and alters the mean bootstrapped distortion ($\bar{d}\left(\theta \right)$) at a given target value *θ* (*N*_boot_ = 1*e*3). **b**. Relative total mean distortion compared between the flat landscape and heterogeneous landscapes (negative values denote reduced distortion for heterogeneous landscape). Red vertical line denotes where the number (and position) of attractors are aligned to peaks in the environmental prior. **c** Schematic of learning in a particle model. Based on the target observed on each trial, the estimated environmental distribution $P_{\text{est}}^{N}\left(\theta \right)=P\left(\theta |\theta_{1:N}\right)$ and the particle landscape $U_{\text{est}}^{N}\left(\theta \right)$ is updated at the target location. Over the course of many trials, the estimated distribution $P_{\text{est}}^{N}\left(\theta \right)$ becomes more similar to the environmental prior *P*_env_(*θ*), and the energy landscape aligns its wells to its peaks. **d** Heatmap showing the landscape updating over the course of many trials. Top trace shows initial landscape. Bottom trace displays the landscape on the final trial. Grey traces are 10 examples of the learning model, black trace is the average learning model’s landscape. **e** Top: *L*^2^−norm for the difference between the experience-dependent belief about the environmental distribution ($P_{\text{est}}^{N}\left(\theta \right)$) and the true environmental distribution (*P*_env_(*θ*)). Bottom: Running average of the learning model’s total mean distortion (${\bar{d}}_{\text{tot}}$). Parameters for all sub-figures as listed in Methods Table 1.

In addition to the form of the energy landscape, mean distortion is impacted by the form of the environmental prior *P*_env_(*θ*). While the conditional probability of responses is only altered by heterogeneity in the energy landscape, the marginal probability of response is impacted by both the energy landscape and the environmental prior (S2 Fig), confirming that the mean distortion changes with the environmental prior. Matching the number and position of energy landscape wells to the peaks in the prior, we find the mean distortion $\bar{d}\left(\theta \right)$ is significantly reduced at common (attractor) locations compared to a model with a flat energy landscape, but shows comparable levels of distortion at rare (saddle) locations (Fig 3A; bootstrapped distortion, *p* < 0.05).

We ask if the total mean distortion typically decreases for periodic energy landscapes Eq (1) as compared to flat landscapes when environmental priors are heterogeneous, and find that it is reduced (relative distortion is negative) as well number increases, especially when the attractor number is matched to the number of peaks in the prior (Fig 3B), though the number of wells does not need to exactly match the number of peaks (S3 Fig) based on the relative contributions of diffusion and drift. Energy landscapes misaligned with the environmental prior (e.g., aligned with rare target locations) generally produced response distributions with higher total mean distortion than aligned models (S4 Fig), confirming that aligning attractors to environmental peaks increases coding accuracy of delayed estimates.

#### Experience-dependent learning in particle models

We next ask whether energy landscapes that model the effects of long-term plasticity can infer a prior based on a long sequence of observations. The effective learning rule assumes subjects sequentially infer the environmental prior from long-term experience: After each trial, the subject’s running estimate of the environmental prior is merged with a likelihood function peaked at the current trial’s target value. This evolving estimate of the prior can be represented in the energy landscape by updating the landscape such that peaks in the prior estimate are encoded by attractors, corresponding to regions of synaptic potentiation in an equivalent neural circuit description (see Methods and Fig 3C). Over many trials, the energy landscape develops attractors aligned with the common feature locations (Fig 3c and 3d), regardless of observation order (S5 Fig). Thus, the experience-dependent updates generate learning of the environmental prior, and the energy landscape reflects better estimates of the environmental structure, which reduces total mean distortion, trending towards the distortion of a particle model assigned an environment-matched energy landscape (Fig 3E). Note, because learning is continuous, the environmental distribution is only sampled but not precisely represented, and there is decay in learning due to long term depression, the stationary profile of the energy landscape does not precisely match that of the static heterogeneous model, accounting for the differences in total mean distortion.

### Subjects’ behavior shows hallmarks of learning

**Fig 4:**
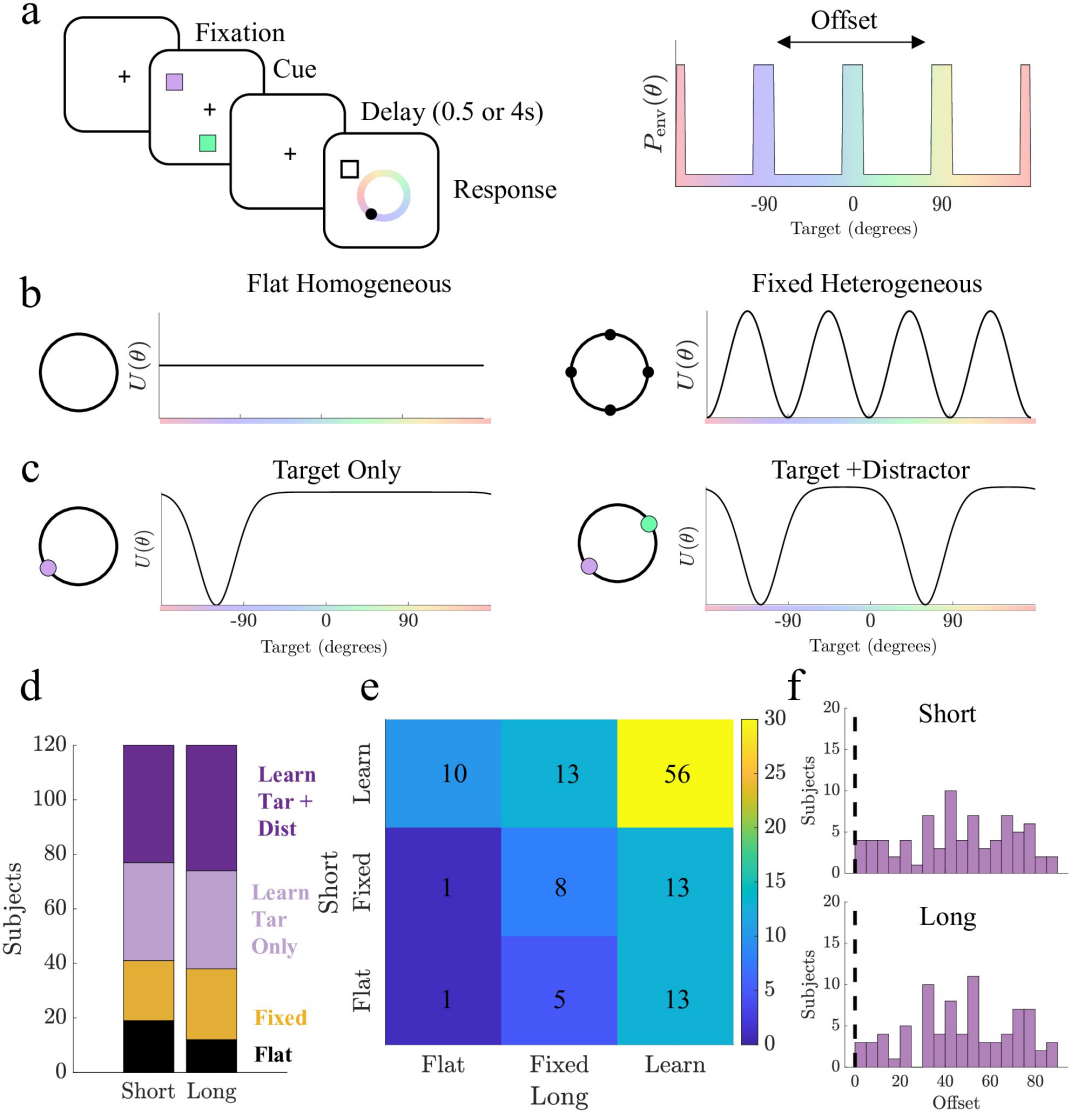
Subject responses based on targets drawn from heterogeneous distributions are best replicated by models governed by heterogeneous landscapes (static and learned). **a**. Experiment 2 from [16] in which subjects were shown two items, each of which could be drawn from a heterogeneous distribution whose peaks were evenly distributed but randomly offset for each subject. Subjects were prompted to respond with the corresponding color of one item. **b**. Fixed particle models used. Homogeneous landscape includes one free parameter, diffusion. Fixed Heterogeneous models include at least three free parameters: amplitude, number of wells, and diffusion. **c**. Learning particle models. Each model updates iteratively based on three parameters: width of the bump, depth of the bump, and diffusion. Target-Only learning models incorporate only the target prompted for response, and Target+ Distractor models incorporate both items. **d**. Number of subjects best matched to each model type displayed in **b** and **c** for short and long delay periods. **e**. Subjects’ best-matched model class for short and long delays. Values show the number of subjects with consistent (upward diagonal) and differing strategy classes across delay times. **f**. Assigned offsets for subjects best matched to Learning models (purple). Dashed lines shows human population bias location.

We next validate our static and learning particle models against responses from a previously reported data set in which 120 human subjects perform sequences of delayed-estimation trials for target colors drawn from distributions along a one-dimensional ring (see [16] for more details). Subjects were cued with two items, the target and distractor, and asked to respond with the color of one item after a short (0.5s) or long (4s) delay. Item colors on each trial were selected from either an (a) uniform stimulus distribution or (b) heterogeneous distribution with four peaks, offset randomly for each subject (Fig 4A).

We ask if subject responses are best described by particle models with energy landscapes from one of three classes: (a) fixed (flat) and uniform; (b) fixed and heterogeneous; or (c) evolving from each subject’s stimulus history. Our fixed and heterogeneous class of models includes three variations: 1. a model with attractors spaced evenly around the ring aligned to each subject’s assigned environmental offset (Static Heterogeneous), with free parameters for the amplitude, the number of attractors, and the noise amplitude; 2. a variation allowing the offset of the attractors to deviate from the peaks of the prior (Offset Heterogeneous model); and 3. a variation in which the energy landscape is determined by two Fourier modes (Dual Heterogeneous model) (Fig 4B and S6 Fig).

We also consider four learning models (Fig 4C): one form (two models) updates the energy landscape based only on the target (Target Only), and another form (two models) updates the potential landscape based on both observed items (Target + Distractor). The initial prior (initial landscape) is also varied to account for subjects’ potential systematic biases, since subjects can exhibit color biases even given uniform environmental priors [16]. Learning models are initialized either with a flat landscape (Flat Prior) or with a landscape with attractors at the locations of the subject population’s biases identified in [16] (Heterogeneous Prior) (S6 Fig). For simplicity, we consider the classes of models (Flat, Fixed Heterogeneous, and Learning) in Fig 4 with additional results for specific model types in S7 Fig.

To identify the model that best matches each subject’s responses, we apply cross-validation based on the mean squared error between subject and simulated responses (see Methods) across many possible parameter sets for each model. To consider the possibility that subjects apply different strategies based on the delay period, we analyze short and long trials separately. Nearly all subjects’ responses (84% of subjects in short trials and 90% of subjects in long trials) are best described by heterogeneous models, with a majority of subjects applying learning models (66% of subjects in short trials and 68% of subjects in long trials; Fig 4d and S7 Fig). More than half of subjects (54%) apply a consistent strategy type between short and long trials, with 86% of consistent subjects using a learning model (Fig 4e). Of subjects that use the same learning model for both blocks (13 subjects), trial-by-trial analysis reveal that learning models are better matched to trial-specific subject responses once learning occurred, with trial-specific model fits showing a strong likelihood over a fixed heterogeneous model in 92% of subjects (S8 Fig).

We find that many subjects best matched to learning and fixed heterogeneous models have assigned environmental prior offsets centered away from the population biases and not uniformly distributed for both short and long trials (*p* < 0.05, two-sample Kolmogorov-Smirnov test) and that the distribution of assigned offsets for learning model subjects is significantly different than that of subjects best fit to fixed heterogeneous models (*p* < 0.05, two-sample Kolmogorov-Smirnov test), with more learning model subjects having an assigned offset that is far from the population biases (Fig 4f and S9 Fig). Considering subjects who consistently used learning models as compared to those who use different models for each delay or consistently use fixed models, we confirm that learning is more prevalent in subjects with assigned offsets further from population biases (S9 Fig). Given that our learning models implement an experience-dependent updating procedure, these findings suggest that many subjects confronted with observations from an environmental prior that differs from their baseline prior learn the new distribution of stimuli based on this sequence of observations.

### Neural mechanism for learning environmental priors

We next study a neural network model capable of implementing experience-dependent inference of environmental priors, comparable to our particle models [22, 45] (see Methods for a demonstration that this model can be asymptotically reduced to our particle models). A neural field model with lateral inhibitory connectivity [29, 46, 47] describes the evolution of neural activity *u*(*x*, *t*) at locations *x* ∈ [−180, 180] corresponding to preferred stimulus value

$$\begin{array}{c} du\left(x,t\right)=[-u\left(x,t\right)+\int_{-180}^{180}w\left(x,y\right)\;f\left(u\left(y,t\right)\right)dy]dt+\epsilon u\left(x,t\right)dW\left(x,t\right)+I\left(x,t\right)dt. \end{array}$$ (3)

Purely distance-dependent and lateral inhibitory synaptic connectivity *w*(*x*, *y*) = *w*_*hom*_(*x* − *y*) is described,

$$\begin{array}{c} w\left(x-y\right)=w_{exc}\left(x-y\right)-w_{inh}\left(x-y\right)=\;\text{exp}\;[-{\left(x-y\right)}^{2}]-A_{\text{inh}}\;\text{exp}\;[-\frac{{\left(x-y\right)}^{2}}{\sigma_{\text{inh}}^{2}}], \end{array}$$

combining both local excitation and broad inhibition, where *A*_inh_ is the strength of inhibition and $\sigma_{\text{inh}}^{2}$ described the inhibitory spread. Since stimuli lie on a ring, we also pose the neural field upon a periodic ring by wrapping *x*, *y* ∈ [−180, 180] so that the difference *x* − *y* should be interpreted as a circular difference. Comparisons with the particle model require converting to radians 180 ↦ *π*. This combination of local excitation and lateral inhibition supports the formation of persistent neural activity bumps when a transient input is presented at a particular location [11, 29, 46]. When synaptic connectivity depends only on the difference between neurons’ stimulus preferences, bumps have no intrinsically preferred positions in the network and lie along a continuous attractor, establishing an unbiased code for delayed estimation of an input stimulus value. Spatial heterogeneity in connectivity breaks this symmetry to create local attractors as in the particle model. This was either prescribed by a fixed periodic presynaptic function *h*(*y*) = *A*_*n*_ ⋅ cos(*ny*), so that *w*(*x*, *y*) = (1 + *h*(*y*))*w*_*hom*_(*x* − *y*) or learned via a slowly evolving function *s*(*y*, *t*) that depends on presynaptic neural activity so *w*(*x*, *y*) = (1 + *s*(*y*, *t*))*w*_*hom*_(*x* − *y*). Such synaptic heterogeneity can also produce [29] or counteract [28] diverse cellular tuning curves, due to the modulating effects of recurrent architecture. The nonlinear *f*(*u*) is a transfer function, *dW*(*x*, *t*) is the increment of a spatiotemporal Weiner process, and *I*(*x*, *t*) is the input representing the cue (See Methods for more details).

**Fig 5:**
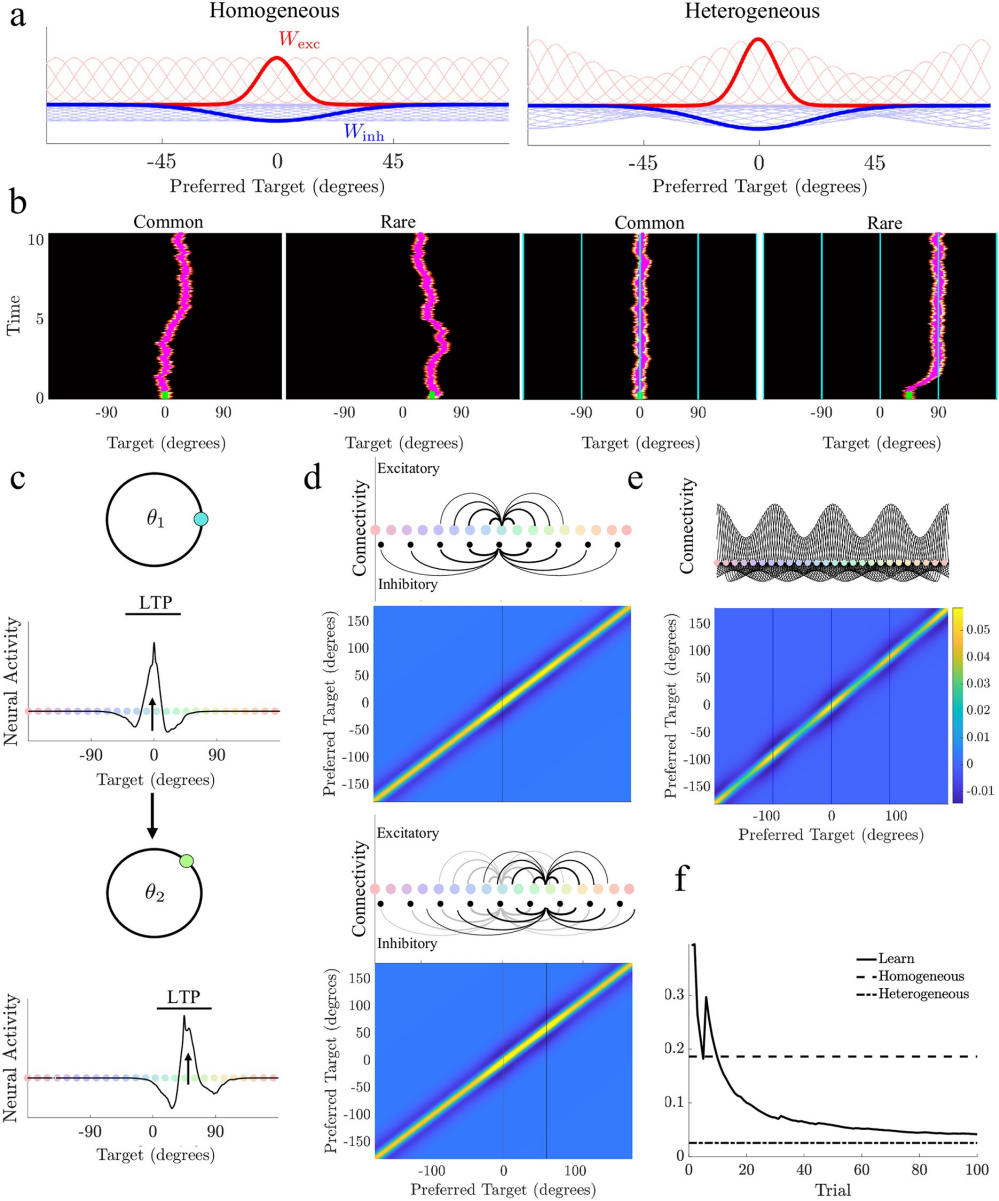
Experience-dependent modulation via long-term potentiation and leak reduces distortion of encoded stimulus values. **a**. Localized excitatory (*W*_exc_) and inhibitory (*W*_inh_) synaptic weights associated with preferred item features in a neural field encoding a delayed estimate. Example of of synaptic weight originating from neuron with preference *θ* = 0 shown in bold. Heterogeneous neural networks are modulated so synaptic footprints originating from peaks of *P*_env_(*θ*) are stronger. **b**. Example bumps of sustained neural activity over 10s delay period originating at common and rare targets in a homogeneous (left) and heterogeneous (right) case. Cyan traces in heterogeneous plots denote attractor locations with enhanced synaptic weights. Stimulus input duration shown in green. **c**. Experience-dependent learning results from pre-synaptic long-term potentiation (LTP) of neurons with a preference for the previous target value. **d**. Learning breaks the symmetry of the spatially-dependent weight kernel, creating enhanced peaks originating from neurons activated across trials. With inactivation over time, connectivity weakens (grey traces from previous trial). **e**. After many trials, the weight matrix recovers the static heterogeneous synaptic structure that matches the current environment. **f**. Average total distortion over time decreases as the experience-dependent neural field model learns the environmental distribution. Parameters for all sub-figures as listed in Methods Table 3.

Strengthening synaptic efficacy at the peaks of the environmental distribution creates attractors (Fig 5A; right) that bias bumps to drift toward the most common stimulus locations (Fig 5B; right). This relationship between the increase in synaptic efficacy and the formation of attractors can be made mathematically precise via direct asymptotic analysis (see Methods). As such, there is a direct relationship between the stochastic dynamics of a bump’s position and the particle models we have discussed already. In short, the introduction of synaptic heterogeneity effectively reshapes an energy landscape that determines the bump position. While this reduces bump wandering when encoding common stimulus values, bumps drift more when instantiated at rare targets, causing larger errors as they are drawn toward attractor locations. As with the particle models, total mean distortion of input stimulus values during the delay is reduced by spatial heterogeneity aligned to the environmental prior (S10 Fig).

We next identify a neuromechanistic learning rule that can modulate synaptic strength based on experience, reshaping the effective energy landscape along which the bump’s position evolves: Synapses emanating from activated neurons (those encoding the stimulus value) are potentiated [22], while a weak decay term reduces synaptic strength in regions that have not recently been observed (Fig 5C). This rule comes from ample evidence for physiological mechanisms supporting long-timescale presynaptic potentiation throughout the nervous system [48, 49]. Such synaptic modulation leads to an increase in connectivity strength at the target location of each trial and a reduction of synaptic efficacy for targets that have not been recently observed (Fig 5D). Updates occur iteratively, so synaptic plasticity modulates weight functions across long timescales to reflect the environmental prior (Fig 5E). As with our particle models, once the neural network learns the environmental prior through experience, it maintains delayed estimates with reduced total mean distortion compared to homogeneous networks with fixed synaptic structure (Fig 5F). Moreover, the weak decay term allows the network to adapt to new heterogeneous environments, updating connectivity to identify the new attractor locations (S11 Fig). Finally, we compared the rate of learning, approximated by the average total distortion over time, between the neural field model and particle models parameterized by subjects who were consistently matched to learning models. We found that the rate of learning between subjects’ best fit particle models and the neural field model were qualitatively the same (S12 Fig). The performance and dynamics of the particle and neural models are thus well aligned.

## Discussion

We have demonstrated that systematic biases observed in human subjects’ delayed estimates can be attributed to environmental experience, specifically corresponding systematic variations in the frequency of stimulus feature values. Our work identifies a potential learning mechanism that can be implemented in reduced models and physiologically motivated neural circuit models and is in line with human response data. This moves beyond prior work, which primarily proposed analogous attractor-based models with fixed energy landscapes [29] and did not propose mechanistic motivations for the attractors [16, 17], by incorporating a Bayesian inference framework and mechanistic basis. Our analysis identifies a mechanism by which sequential inference can be implemented in a particle and neural circuit model with a plausible synaptic plasticity rule.

Beginning with a simplified model of delayed-estimate degradation, we confirm that systematic response biases can be induced by breaking the symmetry of the energy landscape that shapes the evolution of the delayed estimate over time. Such symmetry-breaking stabilizes memories at attractor locations that, when aligned to peaks in the environmental prior, reduce response error at common stimulus values at the expense of larger errors for rare feature values. Overall, total mean distortion of responses is reduced in models with aligned heterogeneous energy landscapes, compared to those with flat landscapes, given the higher propensity of common input stimulus values. Experience-dependent learning of the environment can be implemented neuromechanistically via long-term potentiation that enhances recurrent excitation in neurons encoding common stimulus values and weak synaptic decay for stimuli that have not been observed recently. Responses from human subjects are better matched to models that learn environmental priors than those that are fixed, particularly if the task environment does not well match their baseline beliefs. Thus, subjects confronted with environments that deviate from their priors appear to dynamically update their beliefs based on experience, supporting our hypothesis that systematic biases are learned via experience-dependent plasticity.

Our work supports previous findings showing response variability can be reduced in neuronal networks with spatial heterogeneous synaptic connectivity, even for uniformly distributed stimulus value probabilities [16, 28–30], and extends these findings to determine the efficient tuning of such codes for non-uniform stimulus priors. Our models generate attractive biases whereby delayed estimates tend towards common stimulus values. In related direct perceptual reporting tasks, estimates can be repelled from common stimulus values [50], and such effects have been referred to as “anti-Bayesian” biases. We think one reason for this discrepancy may be that delayed estimation relies on attractor dynamics, which are not necessary in direct reports. In neuronal networks, these attractors are usually created via recurrent architecture, making synaptic heterogeneity and plasticity a plausible mechanism for representing long-term features of the environment. Direct perceptual reports can be modeled by a neural population with no synaptic connectivity, but heterogeneities can be instituted by varying the amplitude, spacing, and width of neural tuning curves across the population [50]. This particular form of model produces anti-Bayesian biases, where responses are repelled from common stimulus locations. It would be interesting to merge these two considerations in future work, to determine how the effects of synaptic and firing rate function heterogeneity combine. The form of systematic working memory biases is quite varied [21, 51–53], as shown in the diversity of best fit models presented in our work, so there are likely multiple neural and synaptic mechanisms that subserve these biases.

For example, subjects’ use of the distractor item as part of their updating procedure suggests that experience-dependent updates could occur during stimulus observation, rather than after subjects’ response as suggested by work on short-term serial biases [8, 22]. Representations of memoranda in multiple item working memory tasks have also been shown to interact, sometimes causing additional errors in memory [18, 54, 55] or reducing cardinal biases [56]. Notably, multi-items were presented sequentially in [56], implying that both short-term plasticity rules [22] and multi-item interactions, such as swapping errors [57], may work in conjunction to produce suboptimal strategies. Future work may consider how multi-item working memory tasks impact experience-dependent learning of task environments.

We had hypothesized that our fixed heterogeneous models would better represent subject responses when environmental priors were more aligned to the population biases (offsets closer to the population bias peaks), because these environments would require less updating to subjects’ environmental beliefs. In contrast, the population of subjects whose strategies are best described by fixed heterogeneous models have a wide range of assigned offsets, while most subjects described by learning models have assigned offsets that deviated from the original population biases. It is unclear whether subjects matched to static models were resistant to learning or were not given sufficient experience (i.e., trials) to adapt. Likewise, it is possible that subjects could apply a dynamic update procedure that is not based on the specific stimuli shown but generally morphs the stimulus distribution to align with the new environment. However, our trial-by-trial analysis of subjects consistently matched to learning models shows that subjects appear to update their environmental distribution in an experience-dependent way. Future studies could investigate more extensively the rate and form of learning when subjects are presented with stimuli drawn from heterogeneous environmental priors to identify the causes for subject-model variability.

Our work has established and validated a novel mechanistic hypothesis to describe how people infer the distribution of environmental stimuli and its impacts on their delayed estimates. Our results support recent findings on training-induced changes in prefrontal cortex [58], suggesting learning over longer timescales can have substantial stimulus-specific impacts in working memory. Moreover, our work posits that limitations and biases in working memory are not necessarily suboptimal, but can be motivated by efficient coding principles and modulated by environmental inference processes. These findings establish a correspondence between environmental inference and working memory that reveals a deeper understanding on the role of working memory in cognitive processes.

## Materials and methods

### Particle model

We described the models here in radian coordinates (i.e., the distance around the ring is 2*π* rather than 360 degrees), but all figures were plotted by rescaling to degrees deg = (180/*π*) · rad. All particle models with fixed energy landscapes used

$$\begin{array}{c} U\left(\theta \right)=-\frac{A_{p}}{n}\cdot \;\text{cos}\left(n\theta \right), \end{array}$$

in which *A*_*p*_ described the amplitude and *n* described the number of wells (attractors). The homogeneous model was recovered when taking *A*_*p*_ = 0. Particle movement was simulated using a stochastic differential equation

$$\begin{array}{c} d\theta \left(t\right)=-U^{\prime }\left(\theta \left(t\right)\right)dt+\sigma dW\left(t\right), \end{array}$$

which incorporated noise as a Wiener process with increment *dW*(*t*). Numerical simulations were performed using the Euler-Maruyama scheme in which the values for *θ* were discretized to 1 degree (*π*/180 radian) bins and time was discretized to 10ms bins. All parameter values listed in Table 1 were used unless otherwise stated.

#### Distortion

The mean distortion for a given input stimulus value *θ* was computed as

$$\begin{array}{c} \bar{d}\left(\theta \right)=\int_{-\pi }^{\pi }P\left(\theta^{\prime }|\theta \right)\left(1-\;\text{cos}\left(\theta -\theta^{\prime }\right)\right)d\theta^{\prime }, \end{array}$$

where *P*(*θ*′|*θ*) indicates the model or subject’s probability of responding *θ*′ given the stimulus was *θ*. Computations are performed using Monte Carlo sampling. To compute stimulus-specific distortion for a given particle model and environment, *θ* was binned and simulations were used to compute $\bar{d}\left(\theta \right)$ for the bin (*N*_sim_ = 10^5^ per bin). Bootstrapping procedures were used to resample distortion and compute the standard deviations (*N*_boot_ = 1*e*3).

Total mean distortion across all stimulus values in an environmental prior was computed

$$\begin{array}{c} {\bar{d}}_{\text{tot}}=\int_{-\pi }^{\pi }\int_{-\pi }^{\pi }P\left(\theta \right)P\left(\theta^{\prime }|\theta \right)\left(1-\;\text{cos}\;\left(\theta^{\prime }-\theta \right)\right)d\theta d\theta^{\prime }, \end{array}$$

which can be approximated by Monte Carlo simulations with initial conditions sampled from the environmental distribution *P*_env_(*θ*) (*N*_sim_ = 10^5^).

#### Conditional and marginal distributions

The conditional probability *P*(*θ*′|*θ*) was computed by simulating the distribution of responses (particle end locations) for each discretized value *θ* (*N*_sim_ = 10^5^). Marginal distributions of the response *P*_resp_(*θ*) were computed by averaging the discrete conditional probability solutions relative to the known environmental distribution *P*_env_(*θ*).

#### Relating the energy landscape to an experience-based posterior

An experience-based posterior can be related to the stationary distribution of a particle on an energy landscape associated with Eq (2). The equivalent Fokker-Planck equation describing the evolution of the distribution *p*(*θ*, *t*) of possible particle positions *θ* at time *t* assuming a potential function *U*(*θ*) was

$$\begin{array}{c} \partial_{t}p\left(\theta ,t\right)=\partial_{\theta }[U^{\prime }\left(\theta \right)p\left(\theta ,t\right)]+\frac{\sigma^{2}}{2}\partial_{\theta }^{2}p\left(\theta ,t\right). \end{array}$$ (4)

We derived the form of *U*(*θ*) that led to a stationary density that corresponds to a particular posterior *L*(*θ*) in the limit *t* → ∞ in Eq (4). The stationary density $\bar{p}\left(\theta \right)$ was analogous to a posterior *L*(*θ*) since it is the probability density that the system represents when there is no information about the current trial’s target remaining. Thus, we derived an association between $\bar{p}\left(\theta \right)$ and *U*(*θ*) to identify how the energy landscape function *U*(*θ*) should be tuned so that $\bar{p}\left(\theta \right)\approx L\left(\theta \right)$. In the limit *t* → ∞, we found Eq (4) becomes

$$\begin{array}{c} \partial_{\theta }^{2}\bar{p}\left(\theta \right)=-\frac{2}{\sigma^{2}}\partial_{\theta }[U^{\prime }\left(\theta \right)\bar{p}\left(\theta \right)], \end{array}$$ (5)

a second order ordinary differential equation with solution

$$\begin{array}{c} \bar{p}\left(\theta \right)=\chi \;\text{exp}\;[-\frac{2U(\theta )}{\sigma^{2}}], \end{array}$$ (6)

where *χ* is a normalization factor. Thus, to match $\bar{p}\approx L$, we need

$$\begin{array}{c} U\left(\theta \right)\approx \frac{\sigma^{2}}{2}\;\text{log}\;\frac{\chi }{L(\theta )}. \end{array}$$

We assumed that we were in the limit of weak heterogeneities, so the deviation of the function *L*(*θ*) from flat will be weak, and $L\left(\theta \right)\approx \frac{1}{2\pi }+\epsilon l\left(\theta \right)$ (where $\int_{-\pi }^{\pi }l\left(\theta \right)d\theta =0$), which allowed us to make a linear approximation

$$\begin{array}{c} U\left(\theta \right)\approx \frac{\sigma^{2}}{2}[\;\text{log}\;2\pi \chi -2\pi l(\theta )]\propto -l\left(\theta \right), \end{array}$$

Thus, we removed the constant shift and were only concerned about the proportionality of the energy landscape to the negative of the variation *l*(*θ*) in the posterior.

#### Experience-dependent particle model

To incorporate learning into the particle model, we updated its energy landscape based on the history of experienced stimulus values according to the equation

$$\begin{array}{c} U_{est}^{N}\left(\theta \right)=\frac{N-1}{N}U_{est}^{N-1}\left(\theta \right)+N_{U}\frac{h-se^{\beta \;\text{cos}\;(\theta -\theta_{N})}}{N} \end{array}$$ (7)

which incorporated a von Mises distribution centered at the location of the true stimulus value *θ*_*N*_ on trial *N* with *s* as the scaling factor, *h* the shift, and *β* the spread. This energy landscape update was meant to represent the trial-by-trial probabilistic update to the stimulus distribution estimate. The mean distortion and the particle landscape were updated iteratively on each trial, such that for each stimulus, the distortion was computed and included in the running average. All parameter values listed in Table 1 were used unless otherwise stated.

The additive update of the particle landscape linearly approximated the typical multiplicative scaling of posterior updating based on successive independent observations. To demonstrate how the updating rule for the energy landscape is related to Bayesian sequential updating of the posterior, recall that that enforcing *U*(*θ*) ∝ −*l*(*θ*) ensured an energy landscape aligned with the learned posterior. Thus, we derived an approximate inferred distribution of possible future stimulus target values, updated based on the observed history *θ*_1:*N*_. We assumed that when an observer sees a target value *θ*_*N*_, they inferred that subsequently similar values are more likely, according to the von Mises distribution

$$\begin{array}{c} f_{\theta_{N}}\left(\theta \right)=Ne^{\beta \;\text{cos}\;(\theta -\theta_{N})}, \end{array}$$

where N was a normalization factor. We noted this was self-conjugate (*f*_*θ*′_(*θ*) ≡ *f*_*θ*_(*θ*′)). We will also assumed that 0 < *β* ≪ 1, so the variation in *f*_*θ*′_(*θ*) was weak, which allowed us to approximate $f_{\theta_{N}}\left(\theta \right)\approx N[1+\beta \;\text{cos}\;(\theta -\theta_{N})]$. Sequential analysis then could determine how a posterior for future observations should be updated based on each observed target. Take *p*_*N*_(*θ*) = *p*(*θ*|*θ*_1:*N*_) to be the posterior based on past observations *θ*_1:*N*_ which can be computed directly as the product of probabilities

$$\begin{array}{c} p_{N}\left(\theta \right)=\frac{\bar{p}}{p(\theta_{1:N})}\prod_{j=1}^{N}\;f_{\theta_{j}}\left(\theta \right), \end{array}$$

where $\bar{p}$ is the uniform distribution and we have utilized the self-conjugacy of *f*_*θ*′_(*θ*) ≡ *f*_*θ*_(*θ*′). We used the linearization of the likelihood function and truncated to linear order in *β* to find

$$\begin{array}{c} p_{N}\left(\theta \right)\approx \frac{1}{2\pi }[1+\beta \sum_{j=1}^{N}\;\text{cos}\;\left(\theta -\theta_{j}\right)], \end{array}$$

which, with the approximate formula for $f_{\theta_{N}}\left(\theta \right)$, can be written as

$$\begin{array}{c} p_{N}\left(\theta \right)\approx \frac{N-1}{N}p_{N-1}\left(\theta \right)+\frac{1}{N}f_{\theta_{N}}\left(\theta \right). \end{array}$$

Lastly, noting the proportional relationship of the desired energy landscape to the posterior, *U*_*N*_(*θ*) ∝ −*l*_*N*_(*θ*), we found that the appropriate update for the energy landscape to match this iterative additive update of the posterior was

$$\begin{array}{c} U_{N}\left(\theta \right)\propto \frac{N-1}{N}U_{N-1}\left(\theta \right)-\frac{1}{N}f_{\theta_{N}}\left(\theta \right), \end{array}$$

which we could rewrite using the full form of $f_{\theta_{N}}\left(\theta \right)$ plus a shift to obtain Eq (7).

Thus, in the long-term limit (as *N* → ∞), the energy landscape convolved the environmental prior *P*_env_(*θ*) against the negative of the likelihood function:

$$\begin{array}{c} U_{\infty }\left(\theta \right)\propto -\int_{-\pi }^{\pi }P_{\text{env}}\left(\theta -\theta^{\prime }\right)\;\text{exp}\;[\beta \;\text{cos}\;\theta^{\prime }]d\theta^{\prime }. \end{array}$$

Given that the environmental prior had the form $P_{\text{env}}\left(\theta \right)=Ne^{A\;\text{cos}(m\theta )}$, we then made the approximation $P_{\text{env}}\left(\theta -\theta^{\prime }\right)\approx \frac{1}{2\pi }+A\;\text{cos}\;\left(m\left(\theta -\theta^{\prime }\right)\right)=\frac{1}{2\pi }+A\;\text{cos}\;\left(m\theta \right)\;\text{cos}\;\left(m\theta^{\prime }\right)+A\;\text{sin}\;\left(m\theta \right)\;\text{sin}\;\left(m\theta^{\prime }\right)$, so we could compute

$$\begin{array}{c} U_{\infty }\left(\theta \right)\propto -A\;\text{cos}\;\left(m\theta \right), \end{array}$$

where $A=A\int_{-\pi }^{\pi }\;\text{cos}\;\left(m\theta^{\prime }\right)\;\text{exp}\;[\beta \;\text{cos}\;\theta^{\prime }]d\theta^{\prime }$ and the other term vanished due to its odd symmetry. This was consistent with the form of the fixed heterogeneity we used to align with this environmental prior.

### Human data

Response data from a delayed estimation task was taken from [16], experiment 2, with permission. The task was administered to 120 consenting subjects with normal color vision in Amazon Mechanical Turk who performed and achieved minimal engagement. Each trial within the task presented a subject with two colored squares simultaneously for 200ms after which time they disappeared and a delay of 500 ms (100 short trials) or 4000 ms (100 long trials) ensued prior to a response being cued by presenting an outlined square in the location of one of the two previous prompt (implicit identification of the target object). Participants then provided an estimate of the cued color by using a mouse to drag a small circle around a ring of colored continuum. Each item had a 50% chance of being drawn from the biased distribution. The biased distribution included 4 peaks spanning 20 degrees, equally spaced about the circle. The offset of the stimulus peaks were picked uniformly and randomly and assigned independently to each subject. The location of the population bias was identified based on the peaks in response frequency across the population of human subjects observed in experiment 1 from [16], which probed subjects to report a color drawn from a uniform distribution but subject showed preferences in the reports.

### Subject model fitting

We fit subject responses to 8 different particle models, separated by trial duration to account for possible changes in strategy for each trial length, and identified the most likely model using cross-validation:

1. **Flat potential** (1 free parameter) in which the particle dynamics were only influenced by diffusion
  $$\begin{array}{c} d\theta (t)=\sigma dW(t). \end{array}$$
2. **Static Heterogeneous** (3 free parameters) in which the particle was subject to drift and diffusion, parameterized by the *A*_*p*_ (amplitude), *n* (number of wells), and noise *σ*,
  $$\begin{array}{c} d\theta \left(t\right)=-A_{p}\;\text{sin}\;\left(n\theta -\theta_{\text{off}}\right)dt+\sigma dW\left(t\right), \end{array}$$
  where *θ*_off_ was the offset assigned to a subject by the experiment (not fit).
3. **Offset Heterogeneous** (4 free parameters) included all of the above parameters but incorporated a free parameter for the offset value $\theta_{\text{off}}^{s}$, such that a subject could use a model not aligned to their assigned offset *θ*_off_.
4. **Dual Heterogeneous** (5 free parameters) assumed that subject response were governed by an energy landscape determined by two frequencies (*n*_1_ and *n*_2_) with amplitudes *A*_1_ and *A*_2_, and assuming the offset to be at the assigned location. The stochastic dynamics of the particle were described
  $$\begin{array}{c} d\theta \left(t\right)=-A_{1}\;\text{sin}\;\left(n_{1}\theta -\theta_{\text{off}}\right)dt-A_{2}\;\text{sin}\;\left(n_{2}\theta -\theta_{\text{off}}\right)dt+\sigma dW\left(t\right). \end{array}$$
5–6. **Target-only Learning** (3 free parameters) assumed the energy landscape was updated on each trial as described in the experience-dependent particle model section above, by adding an inverted von Mises distribution centered at the target location with free parameters for *β* and *s*. Noise was parameterized by *σ* as before. The initial landscape was either chosen to be (a) flat *U*_0_(*θ*) = 0, or (b) heterogeneous $U_{0}\left(\theta \right)=-A_{p}\;\text{cos}\;\left(4\left(\theta -\theta_{\text{off}}\right)\right)$ with *A*_*p*_ = 1 and *θ*_off_ aligned to the offset of the established population biases described above.
7–8. **Target + Distractor Learning** (3 parameters) models were implemented equivalently to the “Target-only” model but were updated by adding two inverted von Mises distributions to the energy landscape on each trial, one centered at the target and the other centered at the distractor stimulus value.

Models were fit to each subject’s set of responses using 5-fold cross-validation performed for short and long delay trials separately. For each cross-validation iteration, we sub-selected a unique 20% of the trials uniformly from across the trial block for testing and used the other 80% of trials for training. For each subject-model, we tested 100 parameter sets, selected randomly from a bounded domain for each parameter (Table 2), on the 80% of the trials (training trials), running 100 simulations with each set of parameters for each trial. The mean squared error (MSE) between each simulated and subject response were computed, and the parameter set with the lowest MSE was selected for that subject-model pair. We then simulated responses for the final 20% of trials using the selected parameter set and computed the MSE for these trials. This process was performed 5 times, testing all trials for a given delay length. The test-set MSEs were then averaged, and the model with lowest mean testing-set MSE was selected for each subject. Our selection of 100 parameter sets was based on the fact that the best-fit parameters were consistent across at least 3/5 cross-validation folds for over 80% of subjects (83% in short trials and 87% in long trials) (S13 Fig). In the case of the learning models, the particle landscapes were updated using all training trials that occurred prior to the trial being simulated, whereas the model parameters were determined based on all of the training trials.

**Table 2:** Parameter ranges for human response model fitting.

Model Class	Variable	Bounded Domain
Flat, Fixed, Learn	*σ*	[0.01, 0.2]
Fixed	*A*_*p*_/*A*_1_/*A*_2_	[0.1, 2]
Fixed	*n*/*n*_1_/*n*_2_	[1, 12]
Fixed	$\theta_{\text{off}}^{s}$	[0, *π*/2]
Learn	*β*	[1, 10]
Learn	*s*	[1, 10]

Trial-by-trial analysis was conducted by identifying subject that were best fit to the same learning model across both delays. Parameters from the cross-validation fold with lowest test-set MSE were used to perform trial-by-trial comparisons between the best-fit learning model and the fixed heterogeneous model. A PDE version of the model fitting procedure was used to extract a precise probability for the subject’s responses, given a particular model and the log likelihood ratio was computed.

### Implementing the neural field model

In the neural field model Eq (3), the firing rate nonlinearity *f*(*u*(*y*, *t*)) was taken to be a Heaviside function

$$\begin{array}{c} H\left(u-\kappa \right)=\{\begin{array}{cc} 1, & u\geq \kappa ,\\ 0, & u<\kappa , \end{array} \end{array}$$

in which *κ* described the firing rate threshold.

Noise *ϵu*(*x*, *t*)*dW*(*x*, *t*) was weak, multiplicative, and driven by a spatially-dependent, white-in-time, Wiener process with the spatial filter that decayed with distance |*x* − *y*|:

$$\begin{array}{c} F\left(x-y\right)=\sqrt{\epsilon }\;\text{exp}\;\left(-|x-y|\right), \end{array}$$

and *ϵ* described the noise strength.

Input to the network corresponding to the true location of the stimulus target at location *x*_targ_ was given by

$$\begin{array}{c} I\left(x,t\right)=I_{0}\left(1-H\left(t-t_{\text{inp}}\right)\right)\;\text{exp}\;[-\frac{{\left(x-x_{\text{targ}}\right)}^{2}}{2\sigma_{\text{inp}}^{2}}], \end{array}$$

where *I*_0_ was the strength of the input, *t*_*inp*_ was the length of time it lasted, and $\sigma_{\text{inp}}^{2}$ parameterized the width of the input. Note, the location *x*_targ_ was sampled from the environmental distribution *P*_env_(*x*) as described above to comprise a long sequence *x*_1:*N*_ across trials.

Neural activity evolved by applying Euler-Maruyama iterations to the timestep *dt* and Riemann integration with *dx* to the integral in the discretized version of Eq (3). The bump’s centroid was then identified as the peak in neural activity at each time *θ*_cent_(*t*) = argmax_*x*∈[−*π*,*π*]_*u*(*x,t*). All model parameters are given in Table 3 and were selected to ensure bumps would not extinguish prior to the end of the delay period. Responses for each trial were reported as the location of centroid at the end of the delay period *θ*_cent_(*T*).

**Table 3:** Neural field parameter values.

Variable	Value
*A*_inh_	0.35
*σ*_inh_	3
*A*_*n*_	0.4
*n*	4
*κ*	0.1
*ϵ*	0.5
*I*_0_	1
*t*_inp_	0.5
*σ*_inp_	1
*T*_*delay*_	10
*A*_*inp*_	1
*s*_*learn*_	0.8
*dt*	0.1
*dx*	0.036
*γ*_*s*_	0.99

#### Linking the neural field and particle models

The dynamics of bump solutions to Eq (3) can be reduced to first order to describe how their position $\tilde{\theta }\left(t\right)$ evolved over time, roughly approximating the centroid (peak location of neural activity). A reduced stochastic differential equation can be derived describing how this position evolves in time due to noise, inputs, and heterogeneity in the weight function. Technical details for such calculation can be found in [22, 45]. Here we give a brief sketch of such analysis, to demonstrate the tight mathematical link between our particle models and the stochastic dynamics of bump solutions to our neural field equations.

Ignoring noise (*ϵ* → 0), heterogeneity (*h*(*y*) → 0), and input *I* → 0, Eq (3) had bump solutions U(x) that satisfied the equation $U\left(x\right)=\int_{-\pi }^{\pi }w\left(x-y\right)f\left(U\left(y\right)\right)dy$ [45]. This bump was marginally stable and lay on a continuous attractor, so it could be placed at any position [−*π*, *π*] [46]. Without loss of generality, we assumed this position was initially *x* = 0, we could track dynamics of the bump’s position $\tilde{\theta }\left(t\right)$ once noise, heterogeneity, and input were reintroduced by deriving a hierarchy of equations for the expansion $u=U\left(x-\tilde{\theta }\right)+\epsilon \Phi \left(x-\tilde{\theta },t\right)+\epsilon^{2}\Phi_{1}\left(x-\tilde{\theta },t\right)+\cdots$. Enforcing solvability of this hierarchy introduced a condition requiring the sum of the noise, input, and heterogeneity to be orthogonal to the nullspace *φ*(*x*) of the adjoint of the operator that comes from linearizing Eq (3) about the bump solution. The result was a drift-diffusion equation whose drift was determined by the energy landscape invoked by both the synaptic weight heterogeneity and input

$$\begin{array}{c} d\tilde{\theta }=-U^{\prime }\left(\tilde{\theta }\right)dt+dW\left(t\right), \end{array}$$

precisely the form of Eq (2), where the drift had contributions from the weight heterogeneity and input

$$\begin{array}{c} U^{\prime }\left(\tilde{\theta }\right)=\underset{\text{heterogeneity}}{\underset{︸}{\frac{\int_{-\pi }^{\pi }\varphi \left(x\right)\int_{-\pi }^{\pi }w\left(x-y\right)h\left(y+\tilde{\theta }\right)f\left(U\left(y\right)\right)dydx}{\int_{-\pi }^{\pi }\varphi \left(x\right)U^{\prime }\left(x\right)dx}}}+\underset{\text{input}}{\underset{︸}{\frac{\int_{-\pi }^{\pi }\varphi \left(x\right)I\left(x+\tilde{\theta },t\right)dx}{\int_{-\pi }^{\pi }\varphi \left(x\right)U^{\prime }\left(x\right)dx}}} \end{array}$$ (8)

and the Wiener process noise W(t) had zero mean and variance

$$\begin{array}{c} \left\langle W{\left(t\right)}^{2}\right\rangle =\epsilon^{2}\frac{\int_{-\pi }^{\pi }\int_{-\pi }^{\pi }\varphi \left(x\right)U\left(x\right)\varphi \left(y\right)U\left(y\right)C\left(x-y\right)dxdy}{{\left[\int_{-\pi }^{\pi }\varphi \left(x\right)U^{\prime }\left(x\right)dx\right]}^{2}}. \end{array}$$ (9)

The heterogeneity and input introduced an energy landscape that steers the position $\tilde{\theta }\left(t\right)$ of the bump as it responds to noise fluctuations. As shown in [22, 45], by dropping the input term and considering a Heaviside nonlinearity *f*(*u*) = *H*(*u* − *κ*), f(U(x))=H(x+a)-H(x-a) and *φ*(*x*) = *δ*(*x* − *a*) − *δ*(*x* + *a*) where *a* was the half-width of the bump such that U(x)>κ for *x* ∈ [−*a*, *a*] and U(x)<κ otherwise and *δ* was a Dirac delta function. As such, we could simplify the energy landscape gradient formula to find

$$\begin{array}{c} U^{\prime }\left(\tilde{\theta }\right)=\alpha \int_{-a}^{a}\left[w\left(y-a\right)-w\left(a+y\right)\right]h\left(y+\tilde{\theta }\right)dy. \end{array}$$

#### Approximation with Fourier modes

Note that by decomposing the even weight function into its Fourier series, we have

$$\begin{array}{c} w\left(x-y\right)=\sum_{k=0}^{\infty }w_{k}\;\text{cos}\;\left(k\left(x-y\right)\right), \end{array}$$

which allowed us to write

$$\begin{array}{c} w\left(a-y\right)-w\left(a+y\right)=2\sum_{k=1}^{\infty }w_{k}\;\text{sin}\;\left(ka\right)\;\text{sin}\;\left(ky\right). \end{array}$$

In a similar way, we could decompose the function describing the heterogeneity in the weight

$$\begin{array}{c} h\left(y\right)=\sum_{k=0}^{\infty }a_{k}\;\text{sin}\;\left(ky\right)+b_{k}\;\text{cos}\;\left(ky\right). \end{array}$$

Approximating by the dominant Fourier mode (assume it is even, *m* = argmax_*k*_*b*_*k*_), we took *h*(*y*) ≈ *b*_*m*_ cos(*my*). Integrating against the difference of the shifted homogeneous weight function, then we found $U^{\prime }\left(\tilde{\theta }\right)\approx 2\alpha_{m}\;\text{sin}\;\left(m\tilde{\theta }\right)$ and thus $U\left(\tilde{\theta }\right)\approx -\frac{2\alpha_{m}}{m}\;\text{cos}\;\left(m\tilde{\theta }\right)$, where

$$\begin{array}{c} \alpha_{m}=\frac{\alpha w_{m}}{2m}\;\text{sin}\;\left(ma\right)\left(\;\text{sin}\;\left(2*ma\right)-2ma\right)+\sum_{k\neq m}\frac{2b_{m}w_{k}}{m^{2}-k^{2}}\;\text{sin}\;\left(ka\right)[m\;\text{cos}\;(ma)\;\text{sin}\;(ka)-k\;\text{sin}\;(ma)\;\text{cos}\;(ka)]. \end{array}$$

Note also that as *m* and *k* differ more, the coefficient in the sum will decrease, suggesting the dominant terms from the series description of *w* will be those for the modes *k* indexed close to *m*. Thus, a scaling of the dominant Fourier mode of the weight heterogeneity well approximated the energy landscape associated with the bump’s stochastic motion.

#### Narrow bump approximation

Assuming the bump width was narrow compared to the length scale of the heterogeneity, we could estimate the integral using the trapezoidal rule

$$\begin{array}{c} U^{\prime }\left(\tilde{\theta }\right)=\alpha a[\left(w\left(2a\right)-w\left(0\right)\right)h\left(-a+\tilde{\theta }\right)+\left(w\left(0\right)-w\left(2a\right)\right)h\left(a+\tilde{\theta }\right)], \end{array}$$

so by expanding the even weight function *w*(2*a*) ≈ *w*(0) + 2*a*^2^*w*″(0) as well as linearizing the heterogeneity $h\left(\pm a+\tilde{\theta }\right)\approx h\left(\tilde{\theta }\right)\pm ah^{\prime }\left(\tilde{\theta }\right)$, we obtained

$$\begin{array}{c} U^{\prime }\left(\tilde{\theta }\right)\approx -4\alpha a^{3}w^{\prime \prime }\left(0\right)h^{\prime }\left(\tilde{\theta }\right), \end{array}$$

and thus

$$\begin{array}{c} U\left(\tilde{\theta }\right)\approx -4\alpha a^{3}w^{\prime \prime }\left(0\right)h\left(\tilde{\theta }\right), \end{array}$$

so the energy landscape generated for the bump position from weight heterogeneity *h*(*y*) was approximately proportional to the negative shape of the heterogeneity. As such, any neurons whose emanating synapses were potentiated/depressed then attracted/repulsed the bump.

#### Plasticity rules in neural field model

Experience-dependent learning was invoked in the neural field model with an evolving pre-synaptic neural modulation so that *w*(*x*, *y*) = (1 + *s*(*y*, *t*)) ⋅ *w*_*hom*_(*x* − *y*), updated each trial *N* based on presynaptic neural activation, $u(y,T_{inp}^{N})$, at the time $T_{inp}^{N}$ when network was stimulated on the *N*th trial in response to the cue at *x*_*N*_. Changes to the pre-synaptic modulation term follow the rule

$$\begin{array}{c} \Delta s\left(y,T_{inp}^{N}\right)=\beta_{s}\cdot u\left(y,T_{inp}^{N}\right)-\gamma_{s}\cdot s\left(y,T_{inp}^{N-1}\right), \end{array}$$

so the first term in the modulation change was potentiation with the profile of neural activity at the time of stimulation on the *N*th trial $T_{inp}^{N}$, with strength *β*_*s*_, and the second term represented the effects of depression during intertrial intervals of inactivity with strength *γ*_*s*_ ∈ (0, 1). Such a rule implemented an activity-dependent form of presynaptic potentiation, which depends only presynaptic activity and affects only synapses emanating from those neurons, deemed transmitter-induced long-term plasticity by [59] and for which multiple mechanisms have been proposed [60–62]. Equivalently, this is a slower form of short term plasticity used in previous neural field models of working memory [22, 38]. As in the case of the energy landscape, we could determine the long-term limiting heterogeneity *s*_∞_(*y*) resulting from the learning rule combined with an environmental prior *P*_env_(*θ*). Approximating the shape of the instantiated bump by a von Mises distribution centered at the location of the stimulus value on each trial and assuming weak modifications to the heterogeneity, the long time limit gave

$$\begin{array}{c} s_{\infty }\left(y\right)\approx \beta_{s}\int_{-\pi }^{\pi }P_{\text{env}}\left(y-y^{\prime }\right)\text{exp}[\beta_{u}\;\text{cos}\;y^{\prime }]dy^{\prime }, \end{array}$$

and, by making the approximation $P_{\text{env}}\left(y-y^{\prime }\right)\approx \frac{1}{2\pi }+A\;\text{cos}\;\left(my\right)\;\text{cos}\;\left(my^{\prime }\right)+A\;\text{sin}\;\left(my\right)\;\text{sin}\;\left(my^{\prime }\right)$, then

$$\begin{array}{c} s_{\infty }\left(y\right)\approx \tilde{\beta }\;\text{cos}\;\left(my\right), \end{array}$$

consistent with the expected form of synaptic heterogeneity and resulting energy landscape.

## Supporting information

S1 Fig Total mean distortion as amplitude and number of wells was varied for two example delay periods. Optimal particle model identified based on minimum mean distortion (magenta dots). (a) Low diffusion (*σ* = 0.01) leads to a optimal models with higher number of wells. (b) Moderate diffusion (*σ* = 0.05) leads to optimal models with a variable number of wells based on amplitude. (c) High diffusion (*σ* = 0.1) leads to a optimal models with lower number of wells.
(TIF)

S2 Fig Comparing energy landscapes (*U*(*θ*)) and heterogeneous feature value distribution (*P*_env_(*θ*)), we find the conditional probability of response *P*_resp,env_(*θ*′|*θ*) and the marginal probability of response *P*_resp_(*θ*) for particle models with homogeneous (a) and heterogeneous (b) (four wells at environmental distribution peaks) landscapes. Parameters as listed in Methods Table 1.
(TIF)

S3 Fig Total mean distortion as amplitude and number of wells was varied for three example delay periods. Optimal particle model identified based on minimum mean distortion (magenta dots). (a) Low diffusion (*σ* = 0.01) leads to a optimal models with higher number of wells. (b) Moderate diffusion (*σ* = 0.05) leads to optimal models with a variable number of wells based on amplitude, often harmonics of the number of environmental peaks. (c) High diffusion (*σ* = 0.1) leads to a optimal models with lower number of wells.
(TIF)

S4 Fig (a) The conditional and marginal probabilities when the heterogeneous particle model has more wells than the environment and offset from the peak locations. This offset leads memoranda to drift to offset locations and shows moderate distortion for all values of *θ*. (b) Total mean distortion in offset heterogeneous particle models as compared to non-offset models for moderate diffusion (*σ* = 0.05). Positive values corresponds with higher levels of distortion in offset models. Parameters: *T*_Delay_ = 1, *n* = 8 offset = 45, all others as listed in Methods Table 1.
(TIF)

S5 Fig Ordering of observations in the learning particle model does not change the shape of the learned landscape or overall distortion. (a) 10 iterations of the learning model with the same observations but randomized permutations produce potential landscapes with the same shape but differing amplitudes. (b) 10 iterations of the learning model with no diffusion (drift only) and the same observations but randomized permutations show the same overall mean distortion after many trials with minor variations in the learning rate. Parameters used: *σ* = 0, all others as listed in Methods Table 1.
(TIF)

S6 Fig (a) All fixed heterogeneous models. Static Heterogeneous model includes three free parameters: amplitude, number of wells, and diffusion. Offset Heterogeneous includes amplitude and number of wells, diffusion, and one additional parameter for offset. Dual heterogeneous considers five parameters: amplitude and number of wells for the first component, amplitude and number of wells for the second component, and diffusion. (b) Learning particle models. Each updates iteratively based on three parameters: width of the bump, depth of the bump, and diffusion. Target-Only learning incorporated only the target prompted for response, and Target+ Distractor incorporated both items. Priors refer to initial landscape, beginning either with a homogeneous (flat) landscape or a heterogeneous landscape that matched the human population biases. Parameter ranges as listed in Methods Table 2.
(TIF)

S7 Fig Subjects’ best matched models for short and long delays. (TIF)

S8 Fig Subject best-fit learning model trial-by-trial analysis. (a) Example of trial-by-trial energy landscape changes (top) and log-likelihood ratio between best learning model compared to the best fixed heterogeneous model (bottom) in subject with the same best-fit learning model for both short and long delays. Red dashed line shows where learning qualitatively overcomes the initial biases in the landscape and corresponds to an increase in LLR trials in favor of the learning model. (b) Total LLR summed across trials for all subjects that were consistently best matched to the same learning model (top) and the fraction of subjects with a total LLR that is positive across trials (bottom). We see that most subjects increase their total LLR over time, suggesting that learning models are becoming more aligned with subjects’ responses.
(TIF)

S9 Fig Histograms of subjects’ offsets for: (a) Fixed heterogeneous subjects for short and long delays. (b) All subjects that were consistently best matched to the same model class or different model classes. (c) Subjects consistently matched to the learning model class or to the fixed heterogeneous class. Yellow denotes fixed heterogeneous class, purple denotes learning class, and blue denotes unclassified.
(TIF)

S10 Fig Total mean distortion in the homogeneous and fixed environment-matched heterogeneous neural field models. Bootstrapped averages (*N*_*Boot*_ = 1*e*3) show a significant decrease in distortion for the heterogeneous synaptic connectivity. All model parameters as listed in Methods Table 3.
(TIF)

S11 Fig Neural field model with initial heterogeneous connectivity that does not match the current environment updates to match the current environmental distribution and decrease distortion. (a) initial connectivity. Black lines denote current environment’s attractor locations. (b) final connectivity scheme. (c) Average total distortion across trials. All model parameters as listed in Methods Table 3.
(TIF)

S12 Fig Average total distortion across all trials for subjects consistently best fit by learning models across both delays (black traces). Plots were created using the parameters with the lowest MSE across all cross-validation folds. Average total distortion from the neural field model (red trace) using the parameters listed in Methods Table 3. The rate that distortion is reduced appears qualitatively similar across the subject-fit particle models and neural field model.
(TIF)

S13 Fig Histograms of the number of consistent parameter matches for a subject’s best-fit model across all 5 cross-validation folds for short and long trials. In short (long) trials, 83% (87%) of subjects were matched to the same parameters 3 or more times.
(TIF)
