# Supplementary figures and images for: Learning efficient representations of environmental priors in working memory
Source: PLoS Comput Biol. 2023 Nov 9;19(11):e1011622. doi: 10.1371/journal.pcbi.1011622 (PMC10662764; doi:10.1371/journal.pcbi.1011622)

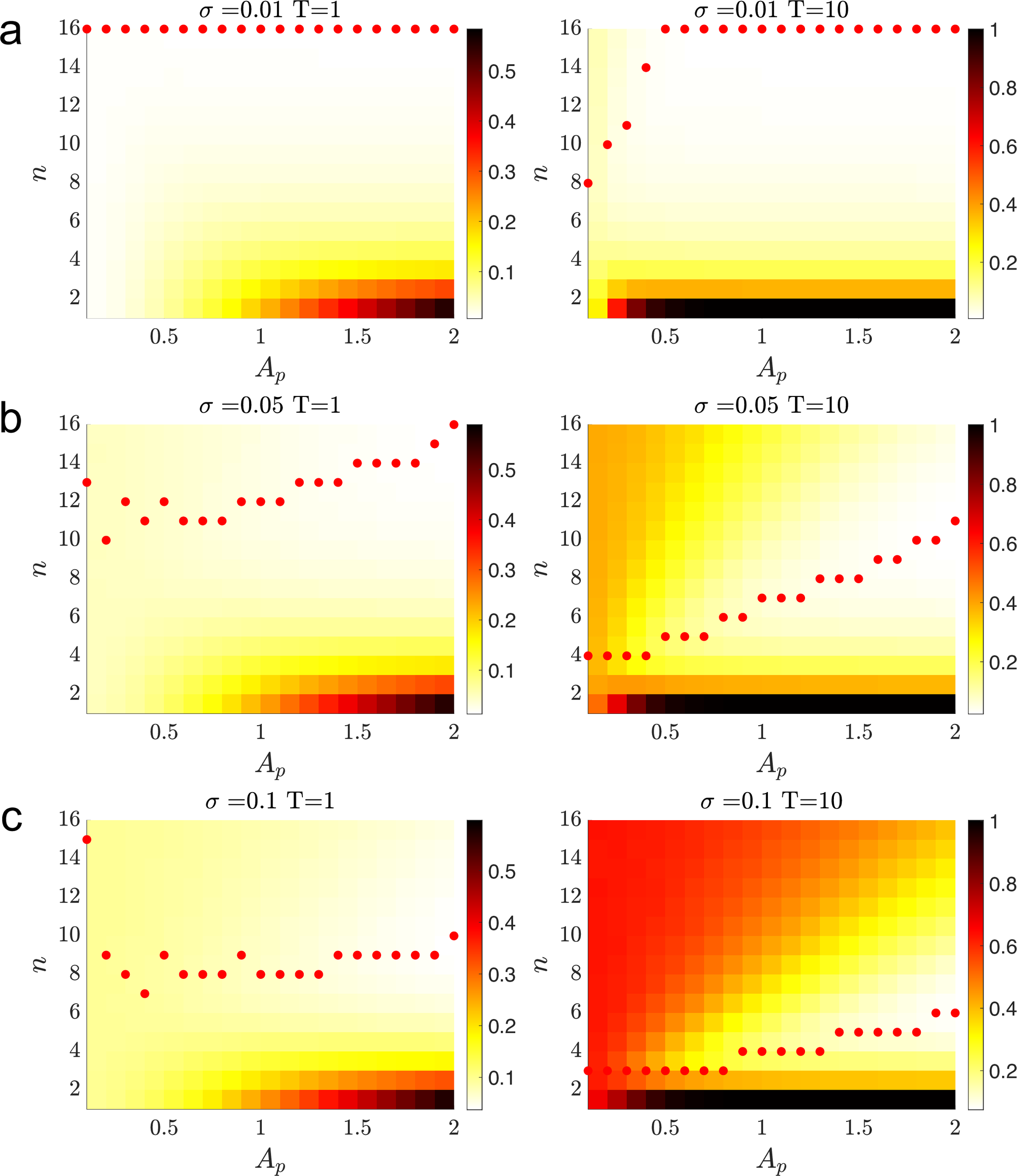

Supplement: S1 Fig — Optimal particle model identified based on minimum mean distortion (magenta dots). (a) Low diffusion (σ = 0.01) leads to a optimal models with higher number of wells. (b) Moderate diffusion (σ = 0.05) leads to optimal models with a variable number of wells based on amplitude. (c) High diffusion (σ = 0.1) leads to a optimal models with lower number of wells. (TIF) [file pcbi.1011622.s001.tif]

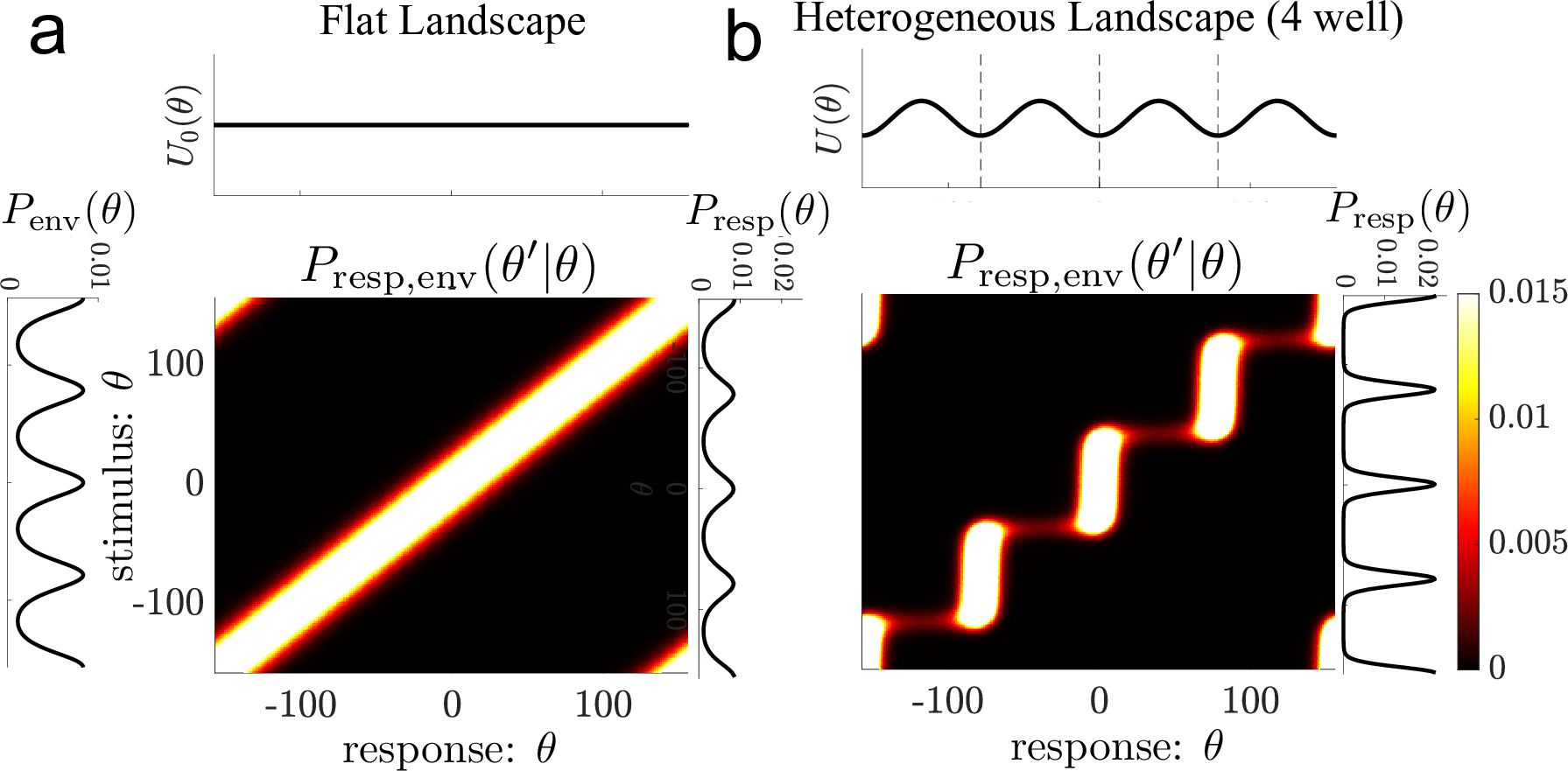

Supplement: S2 Fig — Parameters as listed in Methods Table 1. (TIF) [file pcbi.1011622.s002.tif]

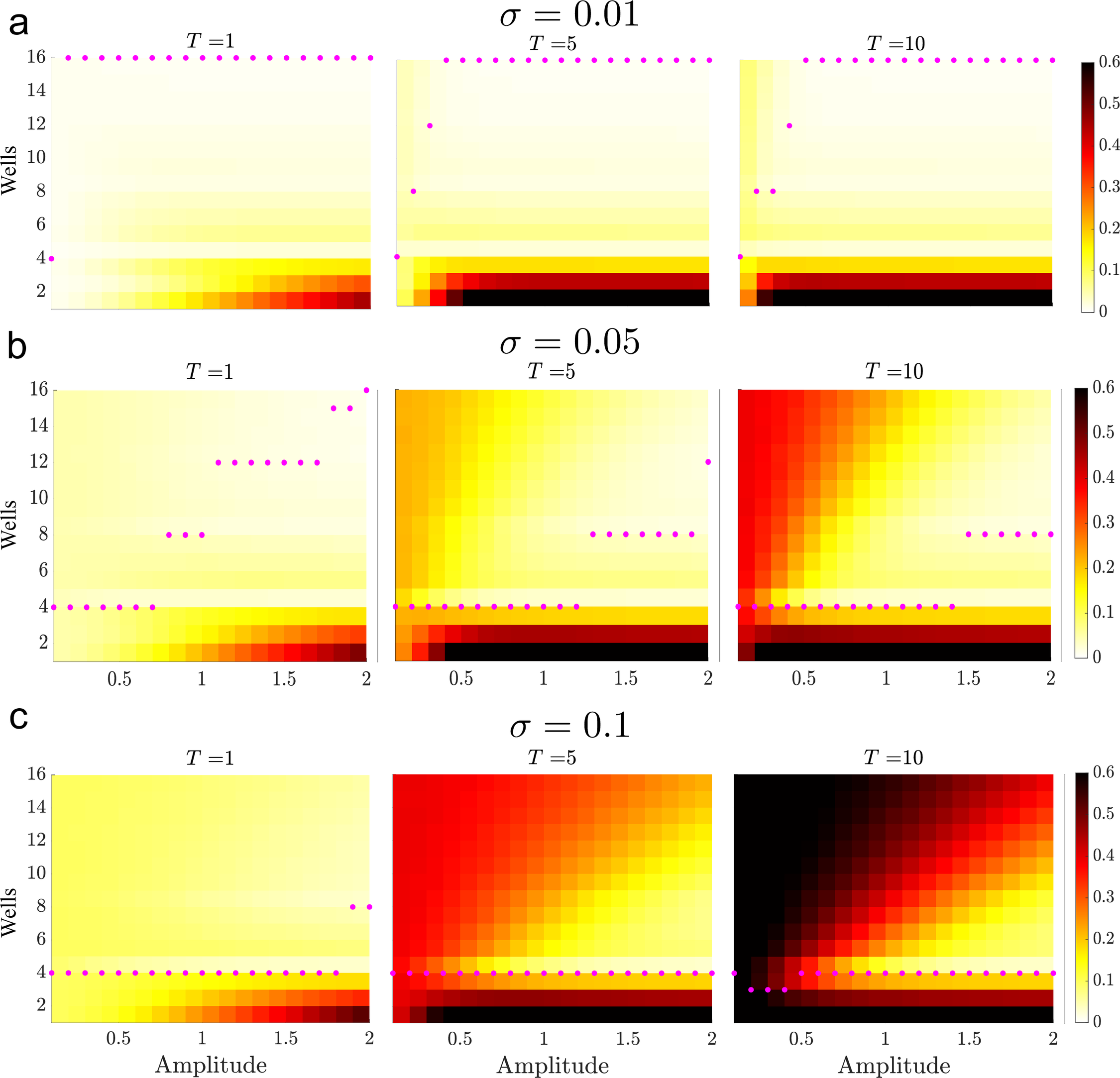

Supplement: S3 Fig — Optimal particle model identified based on minimum mean distortion (magenta dots). (a) Low diffusion (σ = 0.01) leads to a optimal models with higher number of wells. (b) Moderate diffusion (σ = 0.05) leads to optimal models with a variable number of wells based on amplitude, often harmonics of the number of environmental peaks. (c) High diffusion (σ = 0.1) leads to a optimal models with lower number of wells. (TIF) [file pcbi.1011622.s003.tif]

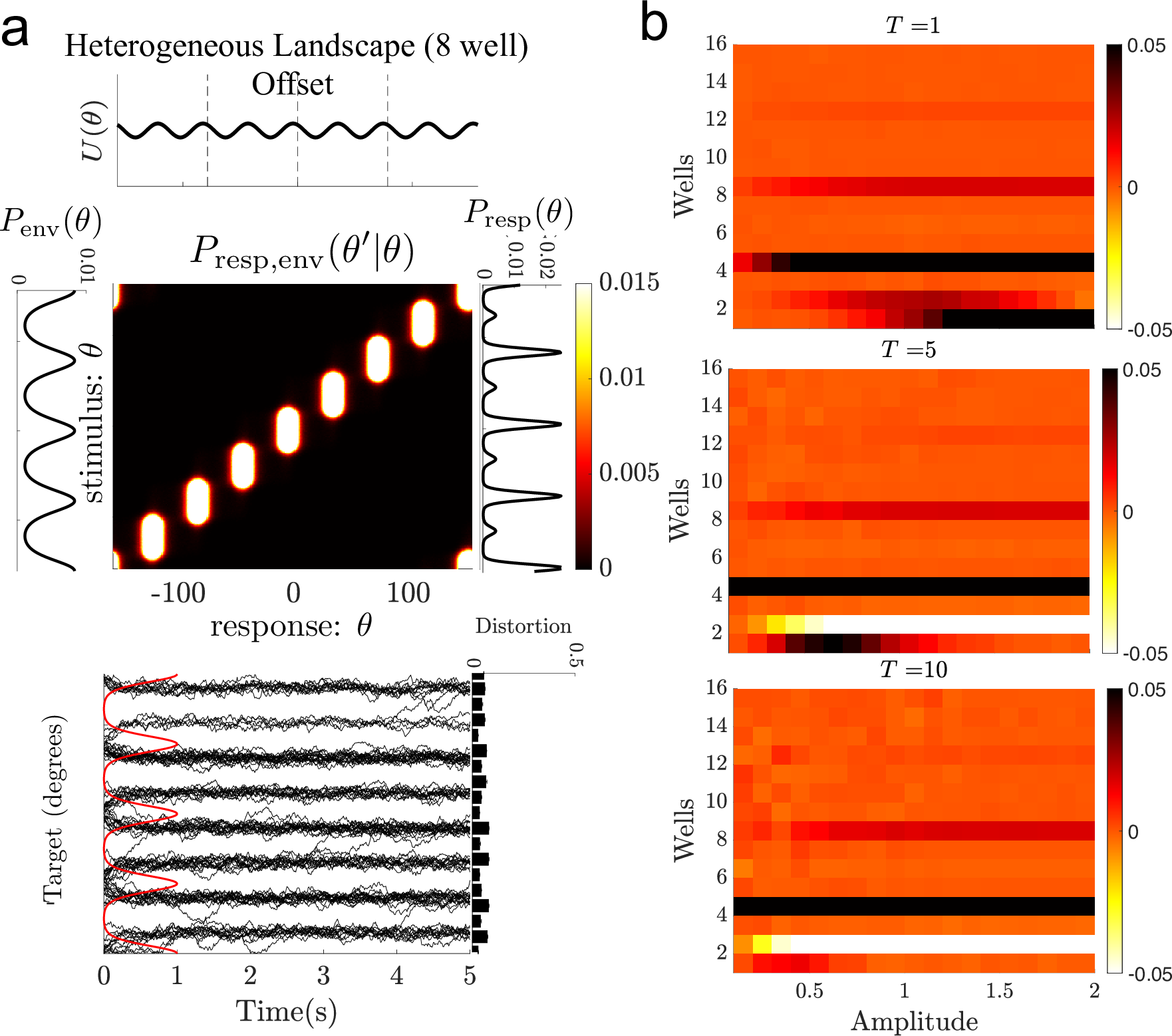

Supplement: S4 Fig — This offset leads memoranda to drift to offset locations and shows moderate distortion for all values of θ. (b) Total mean distortion in offset heterogeneous particle models as compared to non-offset models for moderate diffusion (σ = 0.05). Positive values corresponds with higher levels of distortion in offset models. Parameters: TDelay = 1, n = 8 offset = 45, all others as listed in Methods Table 1. (TIF) [file pcbi.1011622.s004.tif]

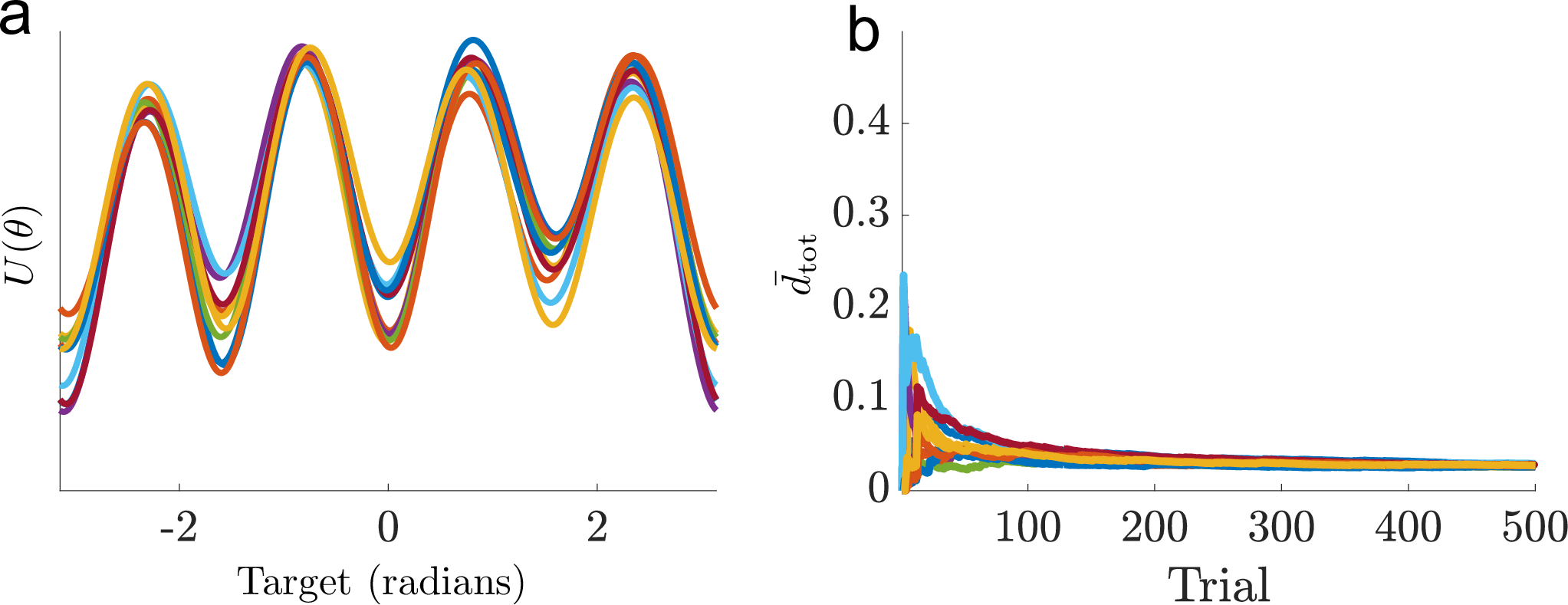

Supplement: S5 Fig — (a) 10 iterations of the learning model with the same observations but randomized permutations produce potential landscapes with the same shape but differing amplitudes. (b) 10 iterations of the learning model with no diffusion (drift only) and the same observations but randomized permutations show the same overall mean distortion after many trials with minor variations in the learning rate. Parameters used: σ = 0, all others as listed in Methods Table 1. (TIF) [file pcbi.1011622.s005.tif]

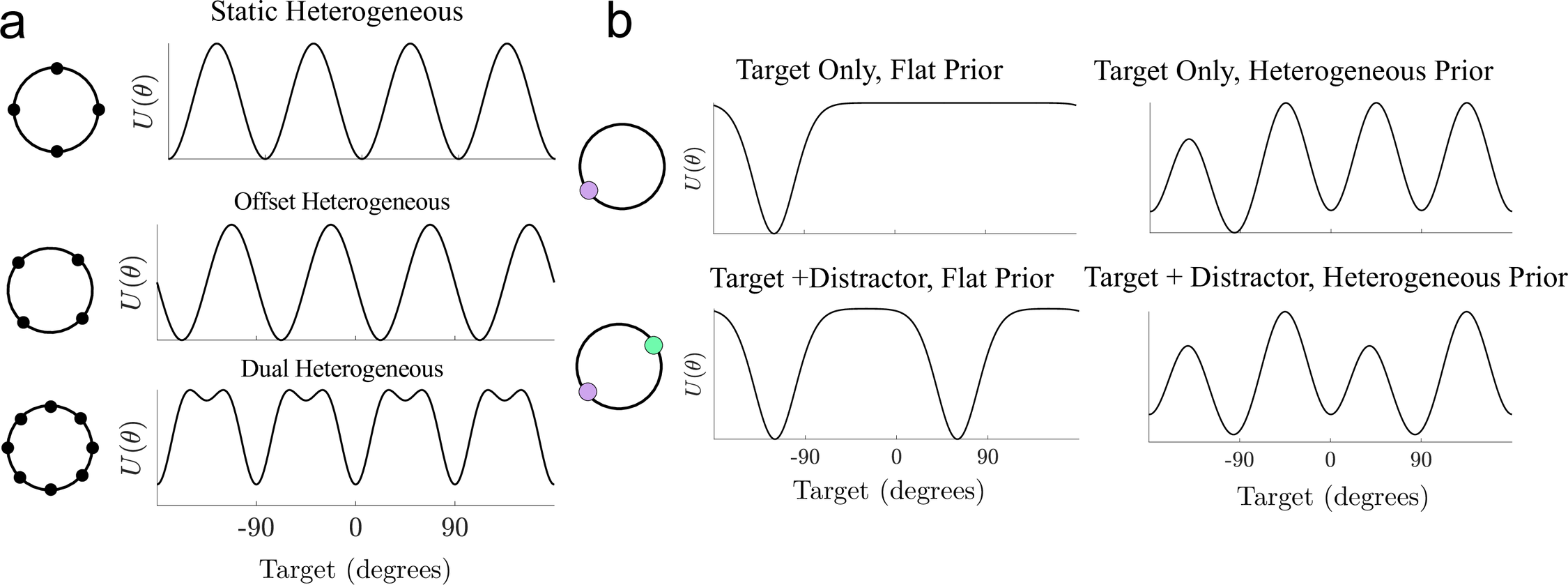

Supplement: S6 Fig — Static Heterogeneous model includes three free parameters: amplitude, number of wells, and diffusion. Offset Heterogeneous includes amplitude and number of wells, diffusion, and one additional parameter for offset. Dual heterogeneous considers five parameters: amplitude and number of wells for the first component, amplitude and number of wells for the second component, and diffusion. (b) Learning particle models. Each updates iteratively based on three parameters: width of the bump, depth of the bump, and diffusion. Target-Only learning incorporated only the target prompted for response, and Target+ Distractor incorporated both items. Priors refer to initial landscape, beginning either with a homogeneous (flat) landscape or a heterogeneous landscape that matched the human population biases. Parameter ranges as listed in Methods Table 2. (TIF) [file pcbi.1011622.s006.tif]

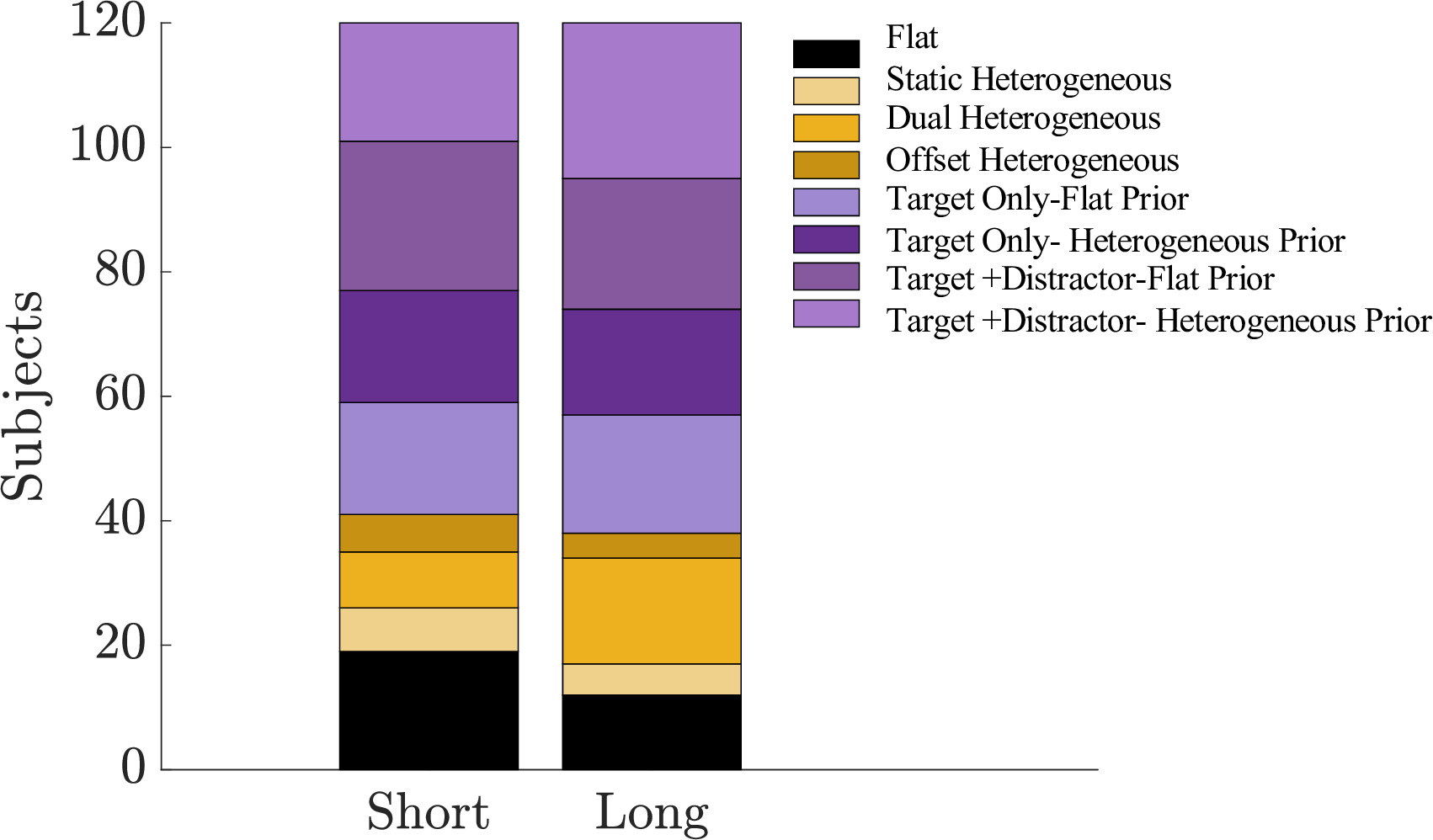

Supplement: S7 Fig — (TIF) [file pcbi.1011622.s007.tif]

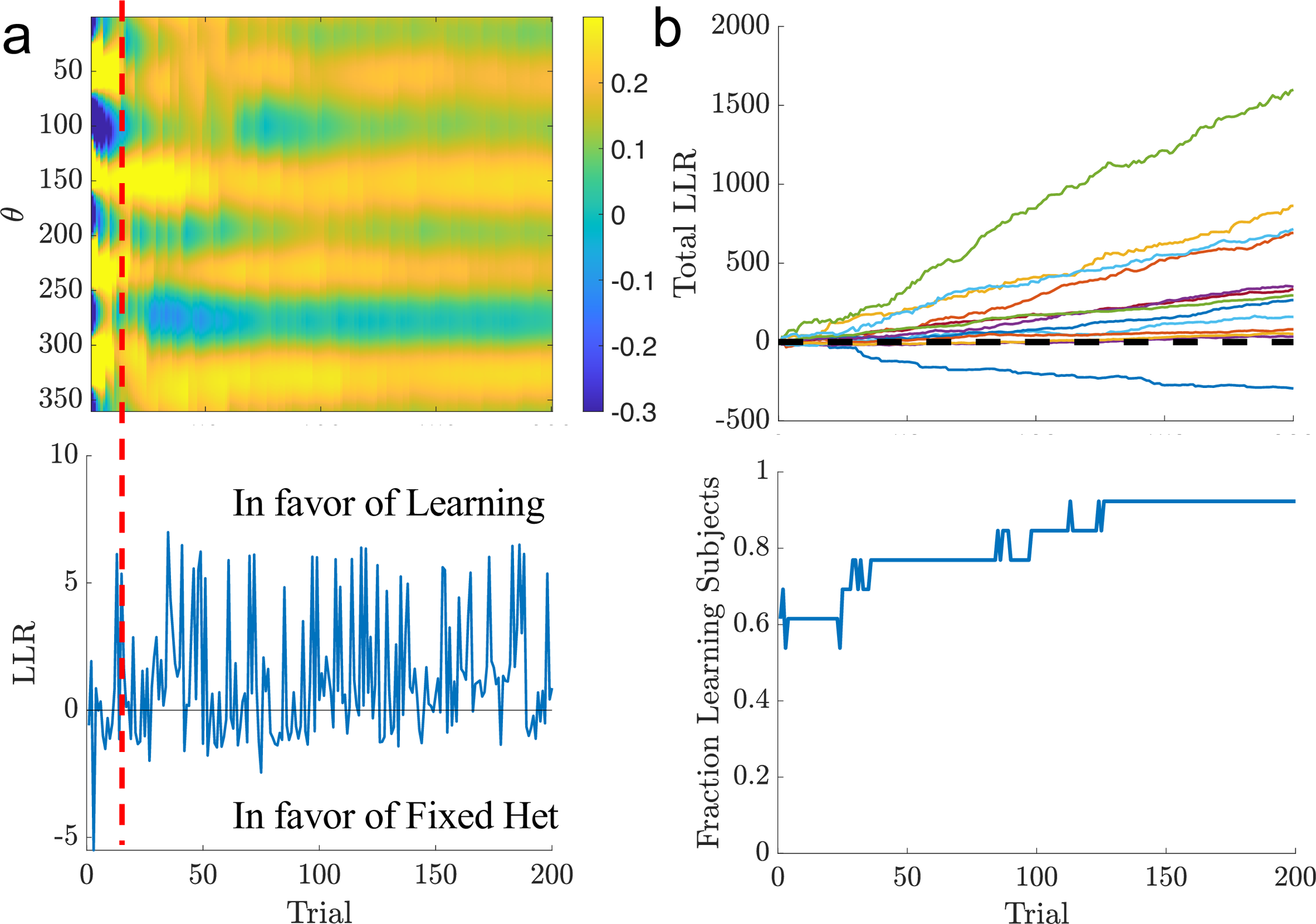

Supplement: S8 Fig — (a) Example of trial-by-trial energy landscape changes (top) and log-likelihood ratio between best learning model compared to the best fixed heterogeneous model (bottom) in subject with the same best-fit learning model for both short and long delays. Red dashed line shows where learning qualitatively overcomes the initial biases in the landscape and corresponds to an increase in LLR trials in favor of the learning model. (b) Total LLR summed across trials for all subjects that were consistently best matched to the same learning model (top) and the fraction of subjects with a total LLR that is positive across trials (bottom). We see that most subjects increase their total LLR over time, suggesting that learning models are becoming more aligned with subjects’ responses. (TIF) [file pcbi.1011622.s008.tif]

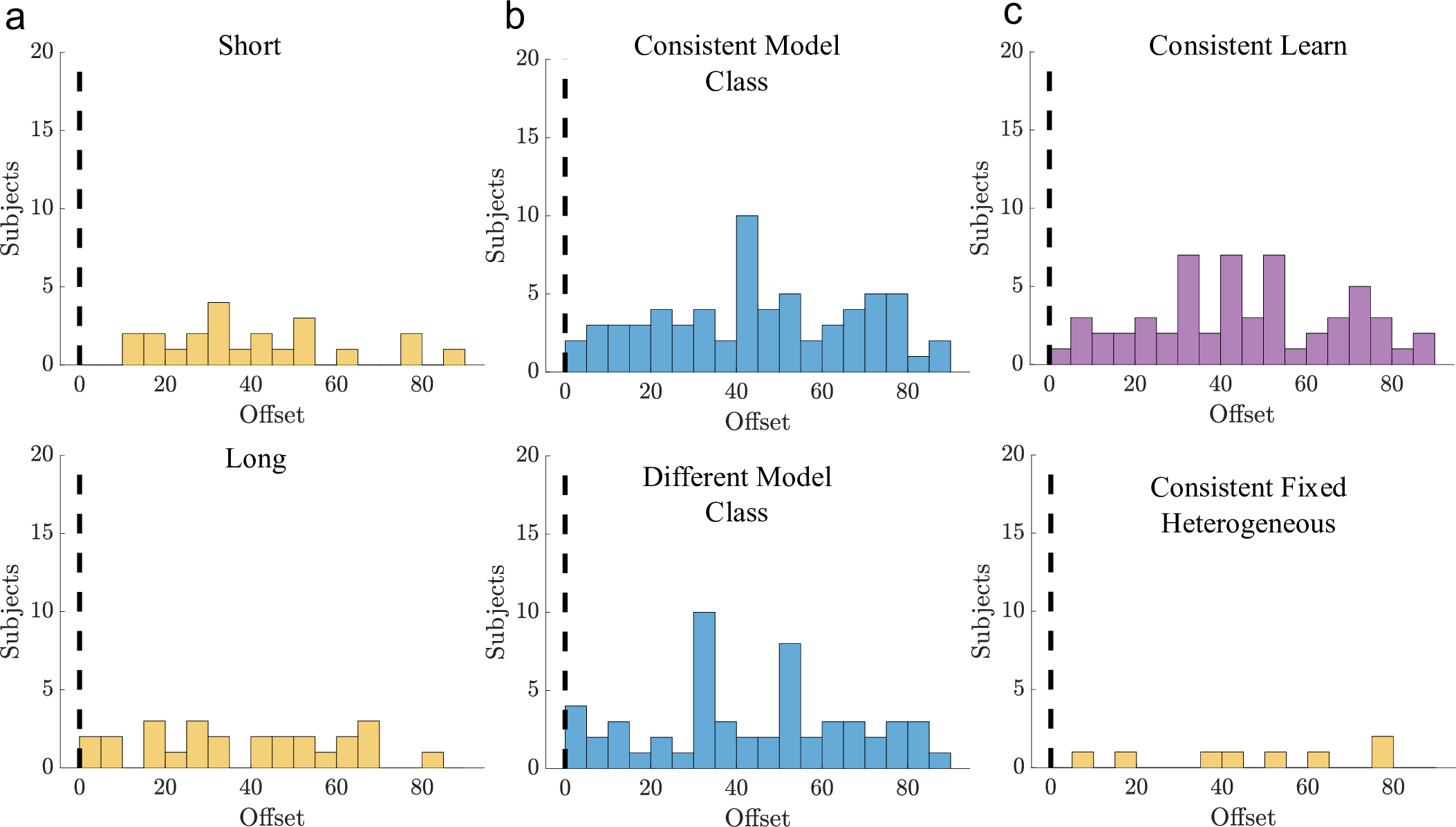

Supplement: S9 Fig — Histograms of subjects’ offsets for: (a) Fixed heterogeneous subjects for short and long delays. (b) All subjects that were consistently best matched to the same model class or different model classes. (c) Subjects consistently matched to the learning model class or to the fixed heterogeneous class. Yellow denotes fixed heterogeneous class, purple denotes learning class, and blue denotes unclassified. (TIF) [file pcbi.1011622.s009.tif]

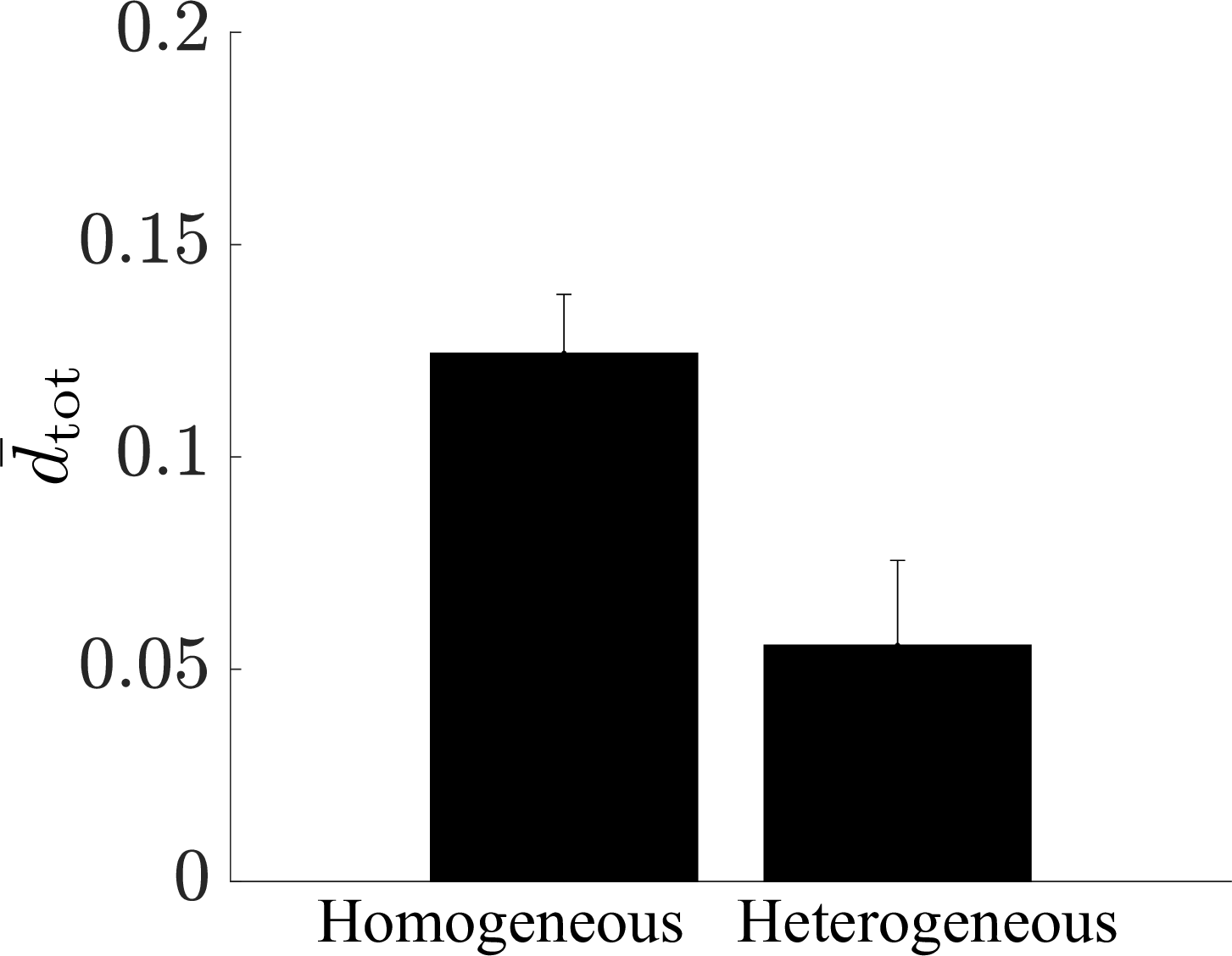

Supplement: S10 Fig — Bootstrapped averages (NBoot = 1e3) show a significant decrease in distortion for the heterogeneous synaptic connectivity. All model parameters as listed in Methods Table 3. (TIF) [file pcbi.1011622.s010.tif]

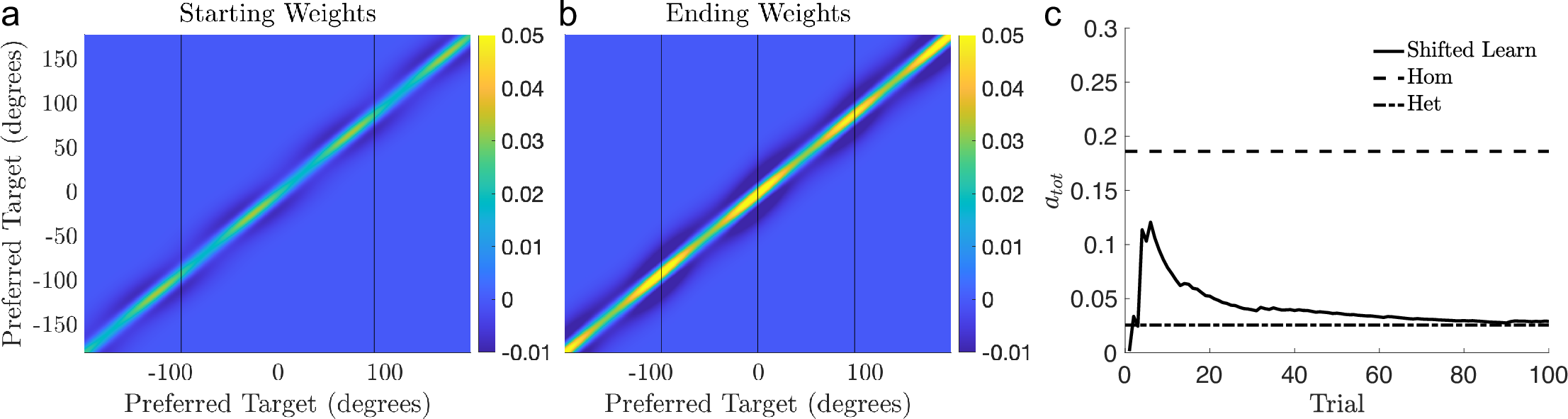

Supplement: S11 Fig — (a) initial connectivity. Black lines denote current environment’s attractor locations. (b) final connectivity scheme. (c) Average total distortion across trials. All model parameters as listed in Methods Table 3. (TIF) [file pcbi.1011622.s011.tif]

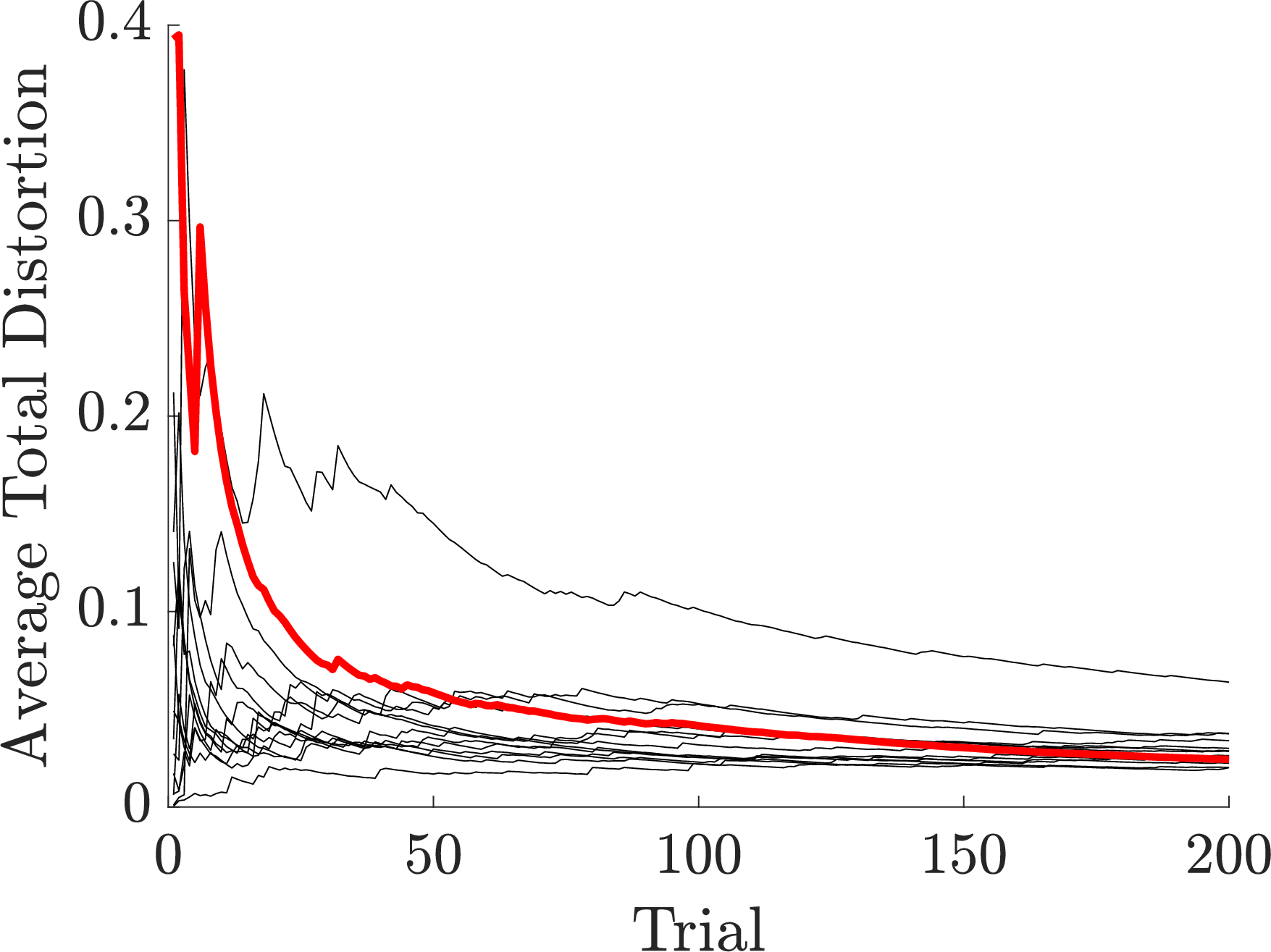

Supplement: S12 Fig — Plots were created using the parameters with the lowest MSE across all cross-validation folds. Average total distortion from the neural field model (red trace) using the parameters listed in Methods Table 3. The rate that distortion is reduced appears qualitatively similar across the subject-fit particle models and neural field model. (TIF) [file pcbi.1011622.s012.tif]

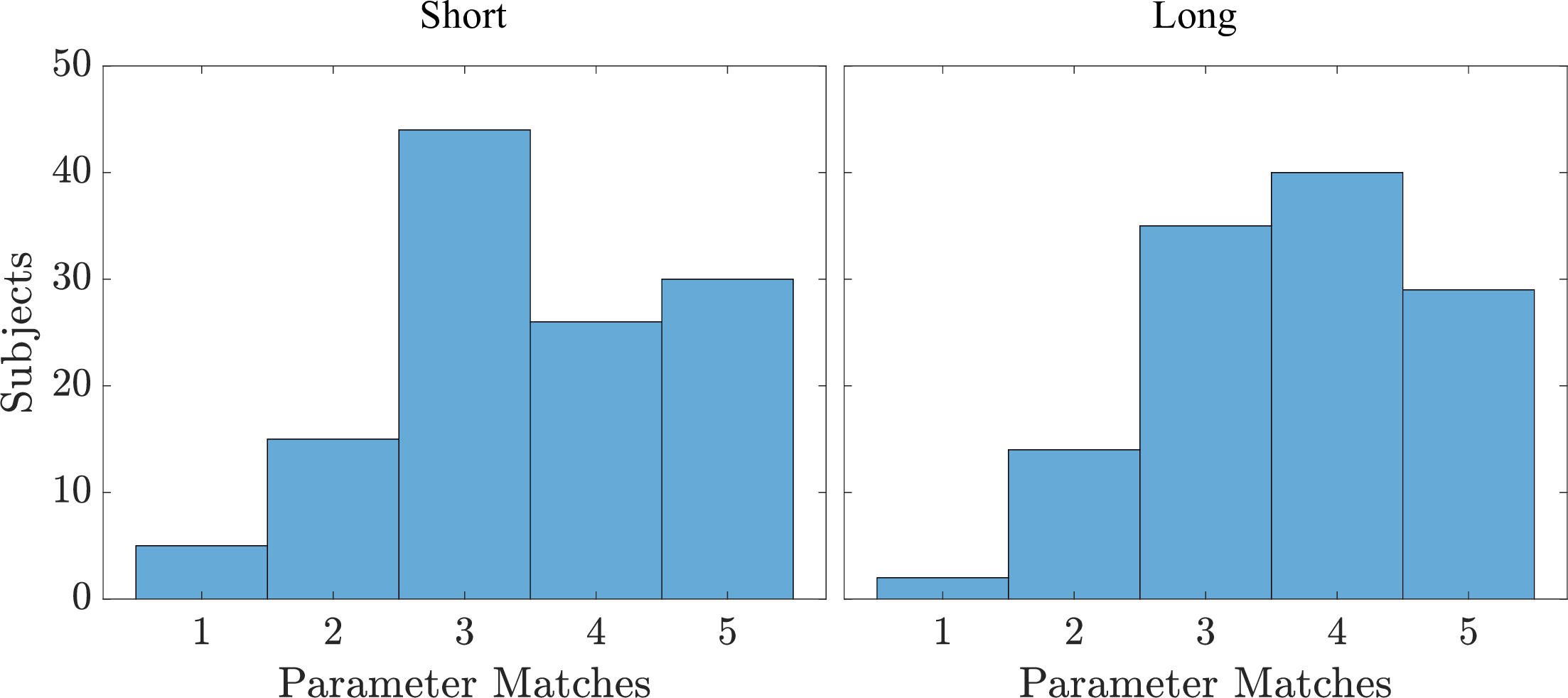

Supplement: S13 Fig — In short (long) trials, 83% (87%) of subjects were matched to the same parameters 3 or more times. (TIF) [file pcbi.1011622.s013.tif]
